# Forecasting shifts in habitat suitability of three marine predators suggests a rapid decline in inter‐specific overlap under future climate change

**DOI:** 10.1002/ece3.9083

**Published:** 2022-07-06

**Authors:** Floris M. van Beest, Rune Dietz, Anders Galatius, Line Anker Kyhn, Signe Sveegaard, Jonas Teilmann

**Affiliations:** ^1^ Department of Ecoscience Aarhus University Roskilde Denmark

**Keywords:** Baltic Sea, climate change, inter‐specific range overlap, marine mammals, MaxEnt, species distribution models

## Abstract

Understanding how environmental and climate change can alter habitat overlap of marine predators has great value for the management and conservation of marine ecosystems. Here, we estimated spatiotemporal changes in habitat suitability and inter‐specific overlap among three marine predators: Baltic gray seals (*Halichoerus grypus*), harbor seals (*Phoca vitulina*), and harbor porpoises (*Phocoena phocoena*) under contemporary and future conditions. Location data (>200 tagged individuals) were collected in the southwestern region of the Baltic Sea; one of the fastest‐warming semi‐enclosed seas in the world. We used the maximum entropy (MaxEnt) algorithm to estimate changes in total area size and overlap of species‐specific habitat suitability between 1997–2020 and 2091–2100. Predictor variables included environmental and climate‐sensitive oceanographic conditions in the area. Sea‐level rise, sea surface temperature, and salinity data were taken from representative concentration pathways [RCPs] scenarios 6.0 and 8.5 to forecast potential climate change effects. Model output suggested that habitat suitability of Baltic gray seals will decline over space and time, driven by changes in sea surface salinity and a loss of currently available haulout sites following sea‐level rise in the future. A similar, although weaker, effect was observed for harbor seals, while suitability of habitat for harbor porpoises was predicted to increase slightly over space and time. Inter‐specific overlap in highly suitable habitats was also predicted to increase slightly under RCP scenario 6.0 when compared to contemporary conditions, but to disappear under RCP scenario 8.5. Our study suggests that marine predators in the southwestern Baltic Sea may respond differently to future climatic conditions, leading to divergent shifts in habitat suitability that are likely to decrease inter‐specific overlap over time and space. We conclude that climate change can lead to a marked redistribution of area use by marine predators in the region, which may influence local food‐web dynamics and ecosystem functioning.

## Graphical abstract

This study estimated species‐specific distribution ranges of three top marine predator species in the Baltic Sea under recent (1997‐2020) and future (2090‐2100) bioclimatic conditions as predicted by the IPCC‐based RCP scenarios (6.0 and 8.5). MaxEnt modelling suggested a marked redistribution of habitat suitability over time and space, driven by divergent species‐specific responses to climate sensitive oceanographic variables. The predicted geographic shifts under the most severe scenario RCP 8.5 will likely lead to a complete loss of inter‐specific overlap of highly suitable habitat, which has important implications for local food‐web dynamics and ecosystem functioning.

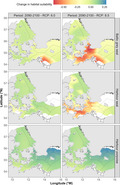

## 1 INTRODUCTION

Climate change threatens biodiversity and ecosystems around the globe (Burrows et al., 2011; Thomas et al., 2004). Rising temperatures and altered precipitation regimes are impacting a wide range of taxa by, for example, changing the suitability of their natural habitat, leading to shifts, contractions, or expansions of species distribution ranges (Chen et al., 2011; Perry et al., 2005). Quantifying and understanding climate change impacts on habitat suitability and species distributions are particularly important for the conservation of marine ecosystems (Doney et al., 2012, Stuart et al., 2021). Marine predators play a crucial role in such climate forecasts because they can integrate information from the bottom to the top of the food web, thereby acting as “sentinels” of an ecosystem's response to climate variability and change (Hazen et al., 2019).

Species distribution models (SDM) and resulting habitat suitability maps are considered valuable tools in ecology and conservation to assess how changing conditions might affect species' distribution ranges (Elith and Leathwick, 2009, Hao et al., 2019, Stuart et al., 2021). SDMs have frequently been used to predict how environmental conditions and climate change may affect future range suitability for a variety of marine species (Robinson et al., 2017) including top predators (Hazen et al., 2012). However, few studies have tried to assess potential changes in habitat suitability of co‐occurring predator species and concomitant shifts in inter‐specific range overlap under contemporary and future conditions (Reisinger et al., 2022). Quantifying spatial and temporal dynamics in species' distributions as well as climate‐induced shifts in spatial overlap is critical to informing management and conservation initiatives, especially in terms of the establishment and management of marine protected areas (Davies et al., 2017). Moreover, estimating shifts in the spatial overlap between co‐occurring species can provide insight into the strength of trophic interactions such as predation and competition (Hunsicker et al., 2013, Orio et al., 2020).

The southwestern Baltic Sea, including the Danish Straits and Kattegat, is home to multiple marine predator species including the Baltic gray seal (*Halichoerus grypus*, Fabricius, 1791), the harbor seal (*Phoca vitulina*, Linnaeus, 1758), and the harbor porpoise (*Phocoena phocoena*, Linnaeus, 1758). The brackish Baltic Sea provides an excellent study system to assess climate‐driven changes in habitat suitability of this predator guild as it is the fastest‐warming semi‐enclosed sea in the world, where sea surface temperatures have increased by circa 1.35°C during the period 1982–2006 (Dutheil et al., 2021), corresponding to seven times the global rate (Belkin, 2009). Projections of future climatic conditions based on the Representative Concentration Pathway (RCP) scenario 8.5 by the Intergovernmental Panel on Climate Change (IPCC) suggest that sea surface temperatures in the southwestern Baltic Sea will increase by an additional 1.35°C, up to approx. 2.7°C compared to 1982, by the end of the 21st century (Saraiva et al., 2019). This would, at the same time, entail a mean expected sea‐level rise of >40 cm (Su et al., 2021). Future climate‐driven changes in sea surface salinity are more complex and uncertain, as sea surface salinity is expected to increase in some areas and decrease in others depending on regional hydrographical conditions (Saraiva et al., 2019).

Our aim was to assess spatiotemporal changes in habitat suitability and inter‐specific overlap among three marine predators co‐occurring in the southwestern Baltic Sea, including the Danish Straits and the Kattegat. Using a machine learning model framework, we estimated and contrasted species‐specific habitat suitability between the periods 1997–2020 and 2091–2100. Candidate predictor variables included a range of gradients in environmental and climate‐sensitive oceanographic conditions within the study area. Given that sea surface temperature and salinity in the southwestern Baltic Sea are important predictors of space use and movements of seals (van Beest et al., 2019) and porpoises (Stalder et al., 2020; van Beest, Teilmann, Dietz, et al., 2018), we expected changes in these dynamic variables to alter future habitat suitability compared to the present situation. In addition, if these species respond differently to future conditions, we expected altered habitat suitability to lead to a redistribution of area use and possibly a change in the spatial overlap between species.

## 2 MATERIAL AND METHODS

### 2.1 Study area & species

**FIGURE 1:**
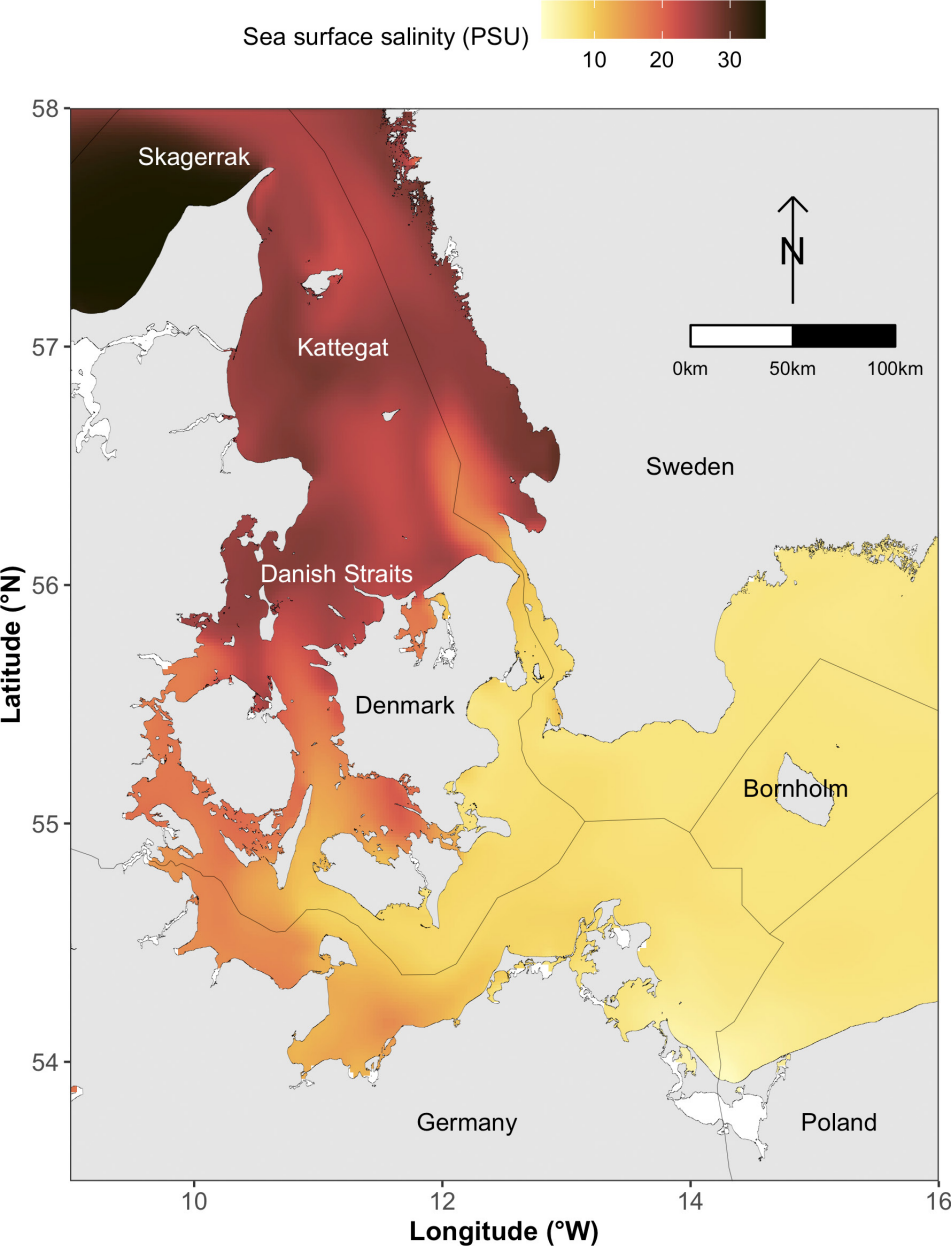
Overview of the study area including the southwestern Baltic Sea, the Danish Straits, and the Kattegat. Also, shown is the sea surface salinity gradient characteristic for the area, which generally declines from north to south due to an inflow of heavier salty water from Skagerrak into the Kattegat, while frontal systems lead to an inflow of brackish surface water from the Baltic Sea into the Danish Straits and Kattegat

The study area covers the southwestern part of the Baltic Sea, including the Danish Straits and the Kattegat (9–16°E, 53.5–58°N: Figure 1). Most of the study area has shallow waters (<60 m) but depths down to 100 m do occur east of Bornholm. The sediment types found in the area are clay, mud, sand, hard bottom complex, and bedrock. Sea surface temperature and salinity vary across seasons but generally decline from north to south due to an inflow of relatively warm (ca. 10°C), salty (ca. 25–30 Practical Salinity Unit [PSU]) water from the North Sea into the Kattegat, while colder (ca. 8°C), brackish (ca. 5–10 PSU) water from the Baltic Sea flows into the Kattegat from the south, causing a complex frontal system in the study area (Pedersen, 1993).

The most abundant marine mammal species in the study area is the harbor porpoise with an estimated population size of ca. 17,000 individuals (Unger et al., 2021). In 2020, a total of ca. 9200 harbor seals were counted in the study area during the molting season (ICES, 2021). Using the correction factor of Härkönen et al. (1999), that would constitute an estimated population size of ca. 16,100 individuals. In 2019, ca. 2500 gray seals were counted during the molt on haulouts in the southern Baltic and Kattegat (Galatius et al., 2020). Assuming that one‐third of the gray seals were at sea during the count, it would constitute an estimated population size of ca. 3750 individuals. The harbor seal and harbor porpoise populations are year‐round residents and use the study area for breeding while the majority of gray seals are visitors that move back into the northern Baltic Proper to breed (Dietz et al., 2015). In addition, genetic studies have shown that Atlantic gray seals (*Halichoerus grypus atlantica*, Nehring, 1886) use the northern part of the study area (Fietz et al., 2016), but none of these individuals have been tagged to track their movements and are therefore not included in the present study. Historically, Baltic gray seals seemed to have been the most abundant seal species in the Kattegat (Olsen et al., 2016).

### 2.2 Location data

**FIGURE 2:**
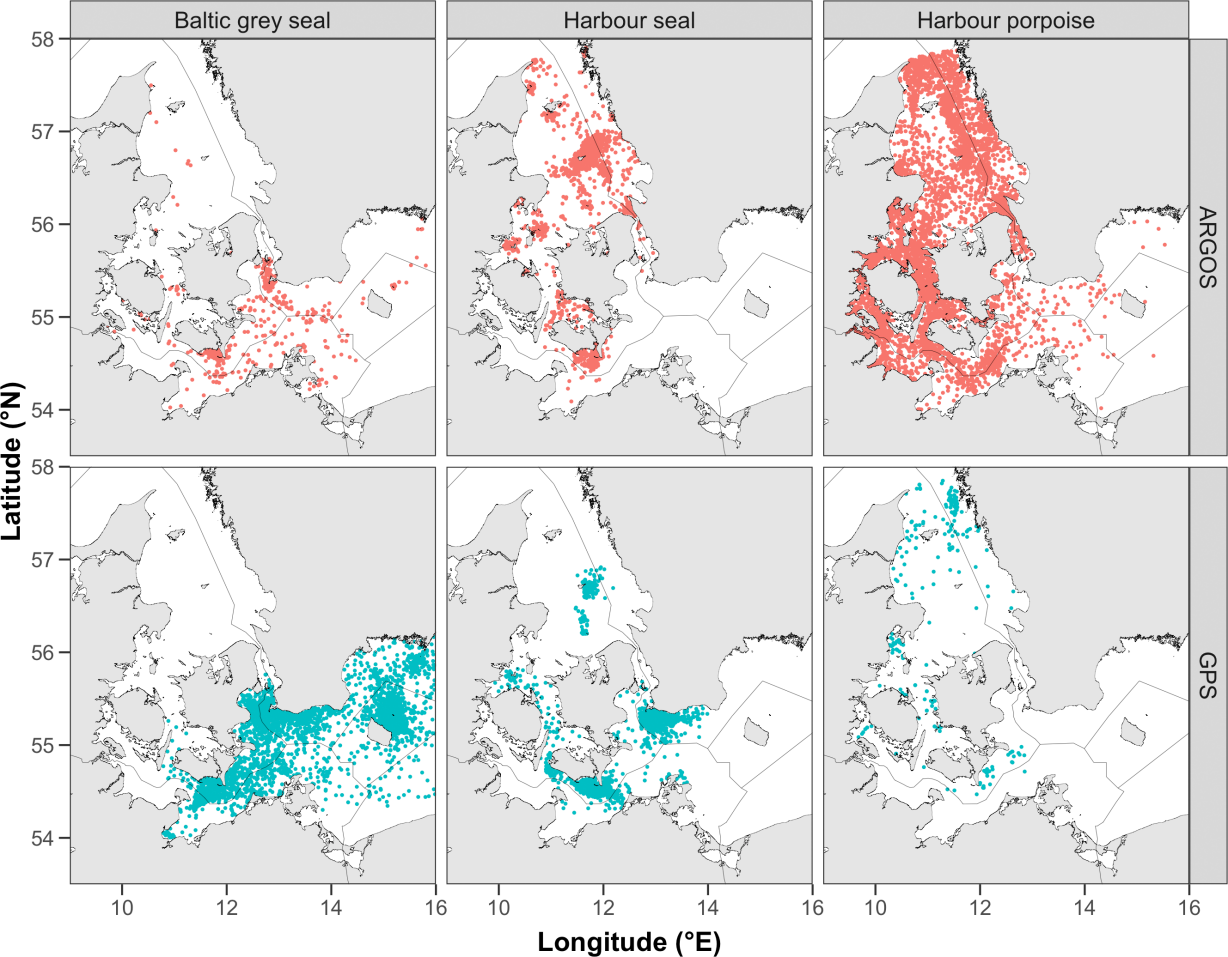
Overview of the location data collected through Argos and GPS tags for each marine predator species collected during 1997–2020 in the southwestern Baltic Sea, including the Danish Straits and the Kattegat

Location data used in this study were collected over the period 1997–2020. Individual harbor porpoises, harbor seals, and Baltic gray seals were fitted with either a Global Positioning System (GPS) tag or an Argos satellite tag to track their movements (Figure 2). All seals were actively captured on haulout sites in Denmark and Sweden, while porpoises were incidentally trapped in pound nets, which are used in near‐shore commercial fisheries in the inner Danish waters. Detailed methods on how individuals were captured, handled, and tagged are described elsewhere (Dietz et al., 2013; van Beest et al., 2019; van Beest, Teilmann, Hermannsen, et al., 2018). Argos tags were programmed to make a limited number of satellite uplinks and acquire a location at predefined times (duty cycles) to increase the battery lifetime. Duty cycles varied with transmission days every 1 and 4 days. GPS tags attempted to acquire and store a location every third min (porpoises) or during each surfacing attempt (seals). In total, location data of 31 Baltic gray seals (13 Argos and 18 GPS), 74 harbor seals (57 Argos and 17 GPS), and 132 harbor porpoises (123 Argos and 9 GPS) were included. Argos satellite tags provide less precise position data than GPS tags and these data were consequently filtered using the Argos‐Filter v7.03 following methods described in Sveegaard et al. (2011). Further pre‐processing of location data included removal of locations collected within 24 h after tagging to reduce behavioral bias caused by capture and tagging (van Beest, Teilmann, Hermannsen, et al., 2018) and removal of positional outliers based on impossible movements (Sveegaard et al., 2011; van Beest, Teilmann, Hermannsen, et al., 2018). Finally, GPS location data were subsampled every sixth hour to reduce autocorrelation (Figure S2.1 in Appendix S2). To this end, we only used locations collected as close as possible to the hours 3:00 a.m., 9:00 a.m., 3:00 p.m., and 9:00 p.m.

### 2.3 Environmental data

We considered a total of seven variables that reflect key environmental and oceanographic characteristics of the study area (for source of data, see Table 1; Figure S2.2 in Appendix S2). Static environmental variables included: “bathymetry (m),” “sea bed slope (°),” and “sediment type (categorical variable including sand, clay, mud, bedrock and hard bottom complex).” The variable “distance to nearest haulout (km)” site was only relevant for harbor and Baltic gray seal models and calculated separately for each species as the Euclidian distance (km) between each location (pixel) within the study area and the closest known haulout site in the region. We used locations of species‐specific haulout sites from Sweden, Denmark, and Germany (Table 1). Distance to nearest haulout was recalculated under future conditions by considering a global mean sea‐level rise, resulting from ice melt and steric rise, of 0.39 m and 0.65 m for RCP scenarios 6.0 and 8.5, respectively (Grinsted, 2015; Katsman et al., 2011; Marzeion et al., 2012). Depending on haulout location, isostatic water‐level rises by 0.10 m in the southwestern Baltic Sea, −0.05 m in southern Kattegat and around Bornholm, and −0.15 m in central and northern Kattegat (Grinsted, 2015; Rosentau et al., 2012) were added to the mean sea‐level rise. Accurate elevation data for seal haulout sites in this area are not known but were based on judgment by two co‐authors (AG and JT) who are familiar with these haulout sites. The forecasted water‐level rises effectively removed some currently available haulout sites from future use (Figure S2.3 in Appendix S2).

**TABLE 1:** Overview of the predictor variables, their units, the original resolution of the raster data, and the source of data download

Variable	Unit	Original resolution	Source^a^ ^,^ ^b^ ^,^ ^c^ ^,^ ^d^ ^,^ ^e^
Bathymetry	m	500 m^2^	HELCOM
Seabed slope	°	500 m^2^	HELCOM
Sediment type	5‐class factor	300 m^2^	HELCOM
Distance to nearest haulout	km	500 m^2^	Denmark
			Sweden
			Germany
Sea surface current velocity	m/s	9.2 km^2^	Bio‐ORACLE
Sea surface salinity	PSU	9.2 km^2^	Bio‐ORACLE
Sea surface temperature	°C	9.2 km^2^	Bio‐ORACLE

*Note:* Prior to MaxEnt model construction, bilinear interpolation was used where needed to ensure that all raster layers had a common spatial resolution of 9.2 km^2^.

^a^ Denmark: Aarhus University.

^b^ HELCOM: https://metadata.helcom.fi/.

^c^ Sweden: Sharkweb https://sharkweb.smhi.se/.

^d^ Germany: Oceanographic Museum, Michael Dähne (pers. comm.)

^e^ Bio‐ORACLE: https://www.bio‐oracle.org.

The dynamic oceanographic variables: “sea surface current velocity (m/s),” “sea surface salinity (PSU),” and “sea surface temperature (°C)” represent averaged monthly values over the years 2000–2014 and projected monthly values over the years 2091–2100 (for both RCP scenarios 6.0 and 8.5). RCP raster data were downloaded from the Bio‐ORACLE database (Assis et al., 2018), which contains joint forecasts of three Global Circulation Models that are part of the CMIP5 collection of model runs used in IPCC's 5th Assessment Report (IPCC, 2013) including CCSM4 (Drake et al., 2005), HadGEM2‐ES (Jones et al., 2011), and MIROC5 (Watanabe et al., 2010). Although these ensembled data incorporate uncertainty in climate change scenarios, it did not allow us to do a quantitative assessment of how different GCMs vary in their projections. We also chose to consider only RCP scenarios 6.0 and 8.5 as these are the most likely future states given current emission rates (Schwalm et al., 2020). The RCP 6.0 scenario represents a high greenhouse gas emission scenario in which total radiative forcing is stabilized after the year 2100, with global mean temperatures projected to rise by about 2.2°C in the year 2100. RCP 8.5 represents a severe emission scenario, with emissions following the same trajectory as during the last decade with global temperatures expected to increase by about 4°C in the year 2100 relative to 1850–1900. We used bilinear interpolation where needed, to ensure that all raster layers had a common spatial resolution of 9.2 km^2^.

### 2.4 Habitat suitability analyses

Habitat suitability of the study species was estimated through the machine learning algorithm maximum entropy (MaxEnt: Phillips et al., 2006, Figure S1.1 in Appendix S1). MaxEnt belongs to a broad class of numerical SDMs that relate occurrence or abundance data with environmental or climatic background data to produce spatially explicit predictions of habitat suitability (Elith and Leathwick, 2009). MaxEnt is particularly suited for presence‐only data with relatively small sample sizes (Elith et al., 2006). We fitted separate MaxEnt models for each species using the procedure outlined below. For more information, we refer to the Overview, Data, Model, Assessment and Prediction (ODMAP) protocol (sensu Zurell et al., 2020) on model development, testing, and evaluation in Appendix S1 in the Supporting Information.

Presence data in the MaxEnt models were species' locations collected in the study area through tagging between 1997 and 2020 (Figure 2). Background points (10000) were randomly sampled for each species and from within the study area. To do so, we first constructed spatial sampling bias files, for each species separately, by computing Gaussian kernel density rasters of all sampling locations (Brown et al., 2017). Sampling bias files (Figure S2.4 in Appendix S2) were subsequently used to increase the likelihood of drawing background points from geographic areas where species occurrences were most common, which is an established method that can lead to more realistic predictions (Merow et al., 2013; Phillips et al., 2009). Both presence and background locations were linked to the environmental raster data. Multicollinearity was assessed by calculating the variance inflation factor (VIF) and Spearman's Rho among the seven predictor variables. Results revealed that VIF <3 and Spearman's Rho <0.6, which suggest that multicollinearity was not of great concern in our data (Dormann et al., 2013). Therefore, we did not adopt a variable selection approach and instead used all predictor variables in the species‐specific models to facilitate comparisons of variable importance and response curves.

To protect against overfitting and to reduce model complexity, MaxEnt uses regularization multipliers (RM) (Phillips et al., 2006). RMs give a penalty for each term included in the model and for higher weights given to a term. Here, we tested different settings of RM using the range 0.5–5.0 in increments of 0.5 for each feature class through the “ENMeval” package in R (Kass et al., 2021; Muscarella et al., 2014). Moreover, we restricted all possible features to “linear,” “quadratic,” and “linear & quadratic” functions to avoid overly complex response curves that would be difficult to explain ecologically. The amount of overfitting for each candidate model was subsequently quantified by calculating the “10% training omission rate” (OR10). OR10 is a threshold‐dependent metric that indicates the proportion of test localities with suitability values (MaxEnt relative occurrence rates) that are lower than the 10% of training localities with the lowest predicted suitability. Omission rates greater than the expectation of 10% typically indicate model overfitting (Muscarella et al., 2014). From the candidate models, we selected the optimal model settings (i.e., RM and feature class) using two sequential criteria (Kass et al., 2021). First, we filtered candidate models with OR10 < 10% and then selected the model with the highest predictive performance as determined by the area under the receiver operating characteristic curve (AUC) value (Table S2.1 in Appendix S2).

Species‐specific habitat suitability maps were created by stacking the raster of the covariates into a multilayered raster and predicting, from the optimal MaxEnt models, the probability of occurrence in each grid cell under both current and future conditions. To ensure that model predictions did not include areas with novel conditions (i.e., conditions for which the model has no training data, thus making predictions unreliable), a multivariate environmental similarity surfaces (MESS) analysis was performed. Following Elith et al. (2010), we used presence locations with associated environmental or oceanographic values under current conditions (1997–2020) as input points and then estimated (dis)similarities in current conditions across the study area extent by comparing to the raster data on future conditions. The MESS analysis was performed for each species and RCP scenario separately. Based on the MESS output, we only retained those areas for model projections where conditions remained similar over space and time (Figure S2.5 in Appendix S2).

### 2.5 Shifts in habitat suitability and inter‐specific overlap

To quantify how changes in environmental and oceanographic conditions may impact the availability of suitable habitats, we contrasted the predicted probability of occurrence, as derived from the complimentary log–log (cloglog) output produced by the optimal MaxEnt models, between the current and future periods. Here, we considered three complementary SDM thresholds (Liu et al., 2013) including Kappa (the value of the probability of occurrence at which Kappa is highest), MSSS (the value of the probability of occurrence at which the sum of the sensitivity (true‐positive rate) and specificity (true‐negative rate) is maximized), and P10 (the value of the probability of occurrence for the lowest 10% of occurrence records). In general, the Kappa threshold was most restrictive as it identified areas with relatively high habitat suitability (probability of occurrence). The MSSS threshold identified areas above a moderate probability of occurrence, while the P10 threshold included most areas above a relatively low probability of occurrence across the study area (Table S2.2 in Appendix S2). For each species and threshold, we computed the absolute change in total area size (km^2^) and the level of clustering (unitless) in habitat suitability between periods. For the latter, we calculated the nearest‐neighbor index (NNI) as a measure of clustering or dispersion (Clark and Evans, 1954). NNI <1 indicates a clustered pattern and NNI >1 suggests dispersion of probability of occurrence.

To assess how shifts in species‐specific habitat suitability might change inter‐specific overlap, we stacked maps depicting highly suitable habitats (i.e., Kappa threshold) for all species, RCP and period combinations, and counted the number of shared raster pixels to compute and estimate changes in the total area size (km^2^) of inter‐specific overlap.

## 3 RESULTS

### 3.1 Predictive performance and variable importance

Predictive performance of the species‐specific MaxEnt models was considered satisfactory with a mean AUC >0.71 for all models and overfitting was considered low with a mean OR10 <0.09 across species‐specific models (Table S2.1 in Appendix S2). The most consistent and important predictor variable influencing habitat suitability across all species was sea surface salinity (PSU), although the response differed between species (see Figure S2.6 for variable importance and Figure S2.7 for response curves in Appendix S2). Habitat suitability of Baltic gray seals was predicted to decline as sea surface salinity increased. A similar response was found for harbor seals, although the negative correlation was less pronounced. In contrast, habitat suitability for harbor porpoises increased with increasing salinity, with a slight decline in habitat suitability at the upper end of the sea surface salinity gradient. Distance to the nearest haulout site was an important variable in predicting habitat suitability for both seal species, with habitat suitability declining strongly with increasing distance from haulout sites. Sea bed slope was an important predictor variable for habitat suitability of harbor seals and porpoises, although the relationship differed between species (negative for harbor seals and positive for harbor porpoises). Sea surface temperature (°C) did not appear to be a highly important predictor variable of habitat suitability across species, although a slight negative correlation was detected for habitat suitability of Baltic gray seals, while harbor seal and harbor porpoise habitat suitability increased slightly with increasing sea surface temperature. The remaining variables included in the species‐specific models had low‐to‐moderate effects on habitat suitability (Figures S2.6 and S2.7 in Appendix S2).

### 3.2 Current and future habitat suitability

**FIGURE 3:**
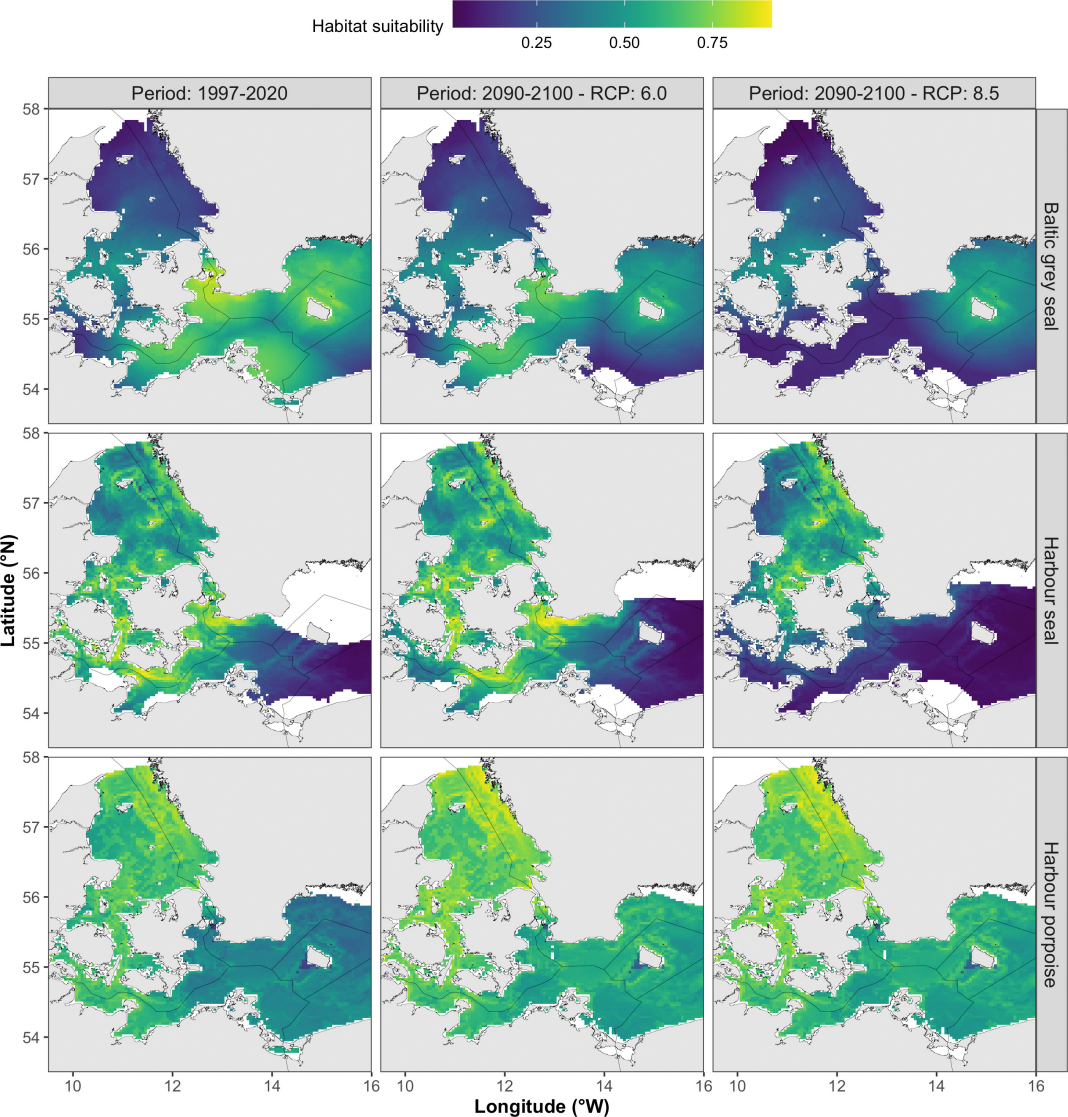
Maps of species‐specific habitat suitability for the periods 1997–2020 and 2090–2100 based on the optimal MaxEnt models using location data collected in the southwestern Baltic Sea. Predicted values are the cloglog output of the species‐specific MaxEnt model with values ranging from 0 to 1 depicted by a blue‐to‐green scale. Note that we did not predict habitat suitability for areas with novel conditions (in white) as identified through species‐specific multivariate environmental similarity surfaces analyses

**FIGURE 4:**
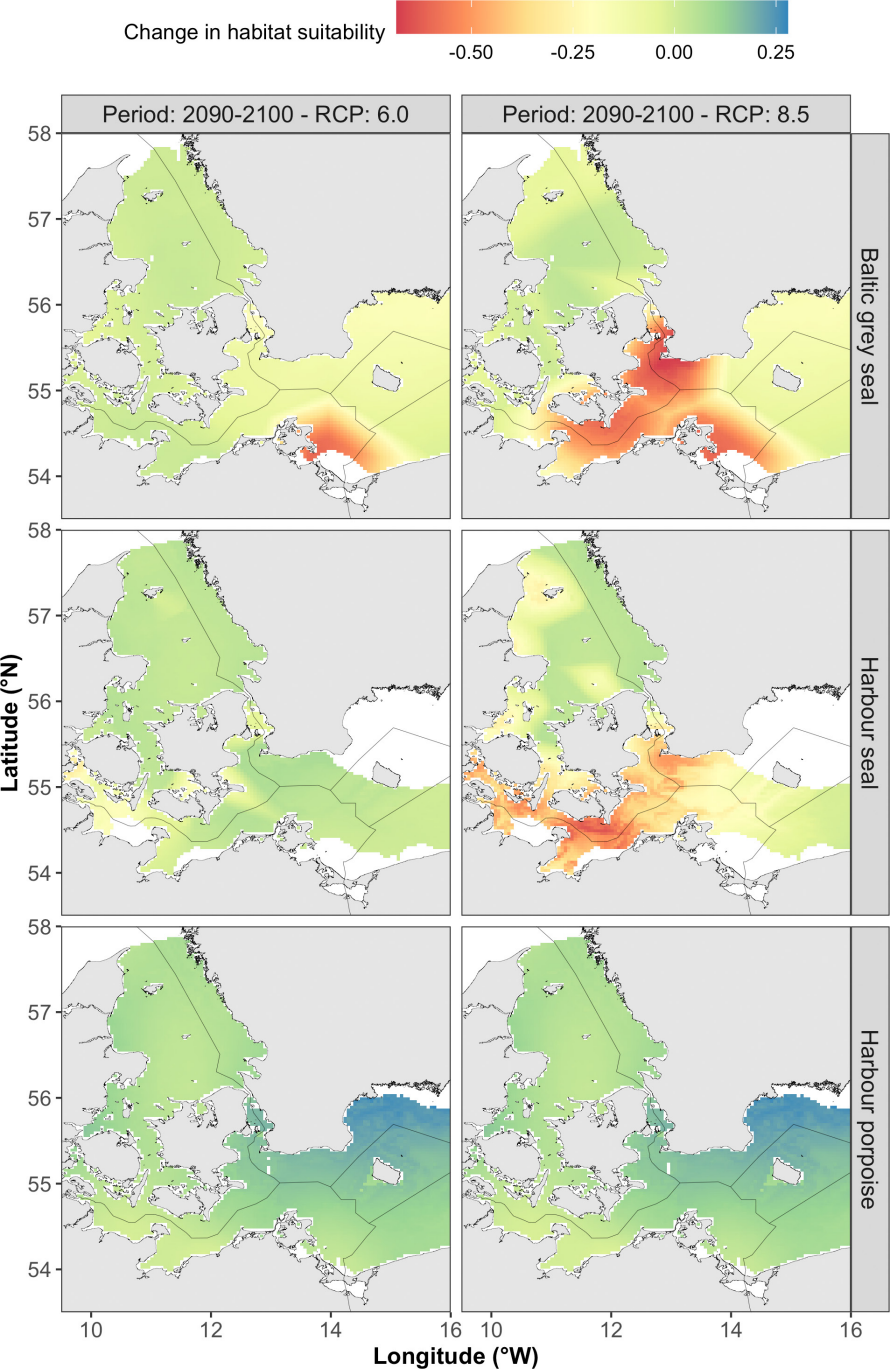
Maps of the predicted spatiotemporal change in habitat suitability for each marine predator species between periods 1997–2020 and 2090–2100 using two RCP scenarios. Areas where habitat suitability was predicted to decrease over time (values <0) are depicted in yellow and red, areas with little change (values ca 0) are indicated in green, while areas where habitat suitability was predicted to increase over time (values >0) are depicted in blue

**FIGURE 5:**
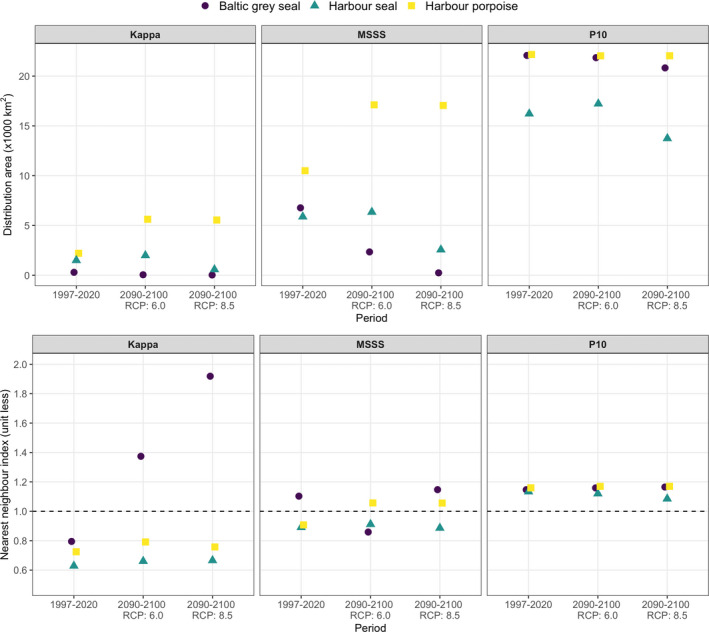
Species‐specific changes in total area size and clustering of habitat suitability within the study area between the periods 1997–2020 (current) and 2080–2100 (depicted by RCPs 6.0 and 8.5). Species are indicated with different colors and symbols as explained in the legend on top. Results are provided for three SDM thresholds: Kappa, MSSS, and P10. Nearest‐neighbor index values <1 indicate a clustered pattern and values >1 suggest dispersion of habitat suitability. Values were derived based on the species‐specific optimal maximum entropy (the complimentary log–log output) models

Spatial mapping of the MaxEnt model results suggested that habitat suitability of Baltic gray seals during 1997–2020 was highest in the mid‐eastern section of the study area (south of the Danish Straits and around Bornholm) and lowest in the northern parts (Kattegat) of the study area (Figure 3). Forecasting of the MaxEnt model results using projected conditions for the period 2090–2100 (Figure 3: RCP scenarios 6.0 and 8.5) and contrasting these with model results of 1997–2020 (Figure 4) suggested that habitat suitability for Baltic gray seals will remain stable and thus low in the northern parts (Kattegat) of the study area, but will decline in the southeastern part of the region under RCP scenario 6.0 and even more so under RCP scenario 8.5. In addition, the availability of suitable habitats for Baltic gray seals was predicted to decline across all SDM thresholds and RCP scenarios as indicated by a reduction in the total area size of suitable habitat over time (Figure 5). While the projected decline in the availability of highly suitable habitats (Kappa threshold) was also predicted to become much more dispersed over space, no such pattern was found for the SDM thresholds MSSS and P10 (Figure 5).

Habitat suitability of harbor seals during 1997–2020 was relatively patchy yet high throughout the study area, except in the southeastern parts toward Bornholm (Figure 3). Forecasting model results for harbor seals under scenario RCP 6.0 suggested that habitat suitability will remain stable over space and time (Figures 3 and 4). Under scenario RCP 8.5, however, harbor seal habitat suitability was forecasted to decline throughout most of the area, especially in the southern Danish waters (Figure 4). Indeed, the total area size of available habitat for harbor seals tended to increase slightly between 1997–2020 and 2090–2100 under scenario RCP 6.0, but decline under scenario RCP 8.5, a pattern that was consistent across SDM thresholds (Figure 5). Model results did not suggest striking changes in the spatial clustering of suitable habitats for harbor seals for any of the SDM thresholds (Figure 5).

Habitat suitability of harbor porpoises during 1997–2020 was highest in the northern parts (Kattegat) of the study area and gradually declined toward Bornholm in the southeastern part of the study area (Figure 3). Forecasting model results for harbor porpoises over the period 2090–2100 suggested that habitat suitability will either remain the same (most of the study area) or increase (north of Bornholm), a pattern that was consistent across RCP scenarios (Figures 3 and 4). Moreover, MaxEnt model output suggested a substantial increase in the availability of high (Kappa threshold) and medium suitable habitats (MSSS threshold), while area size of low habitat suitability to harbor porpoises will remain stable (P10 threshold; Figure 5). Similar to the harbor seal results, model results did not suggest marked changes in the spatial clustering of habitat suitability for harbor porpoises (Figure 5).

### 3.3 Shifts in inter‐specific overlap of habitat suitability

**FIGURE 6:**
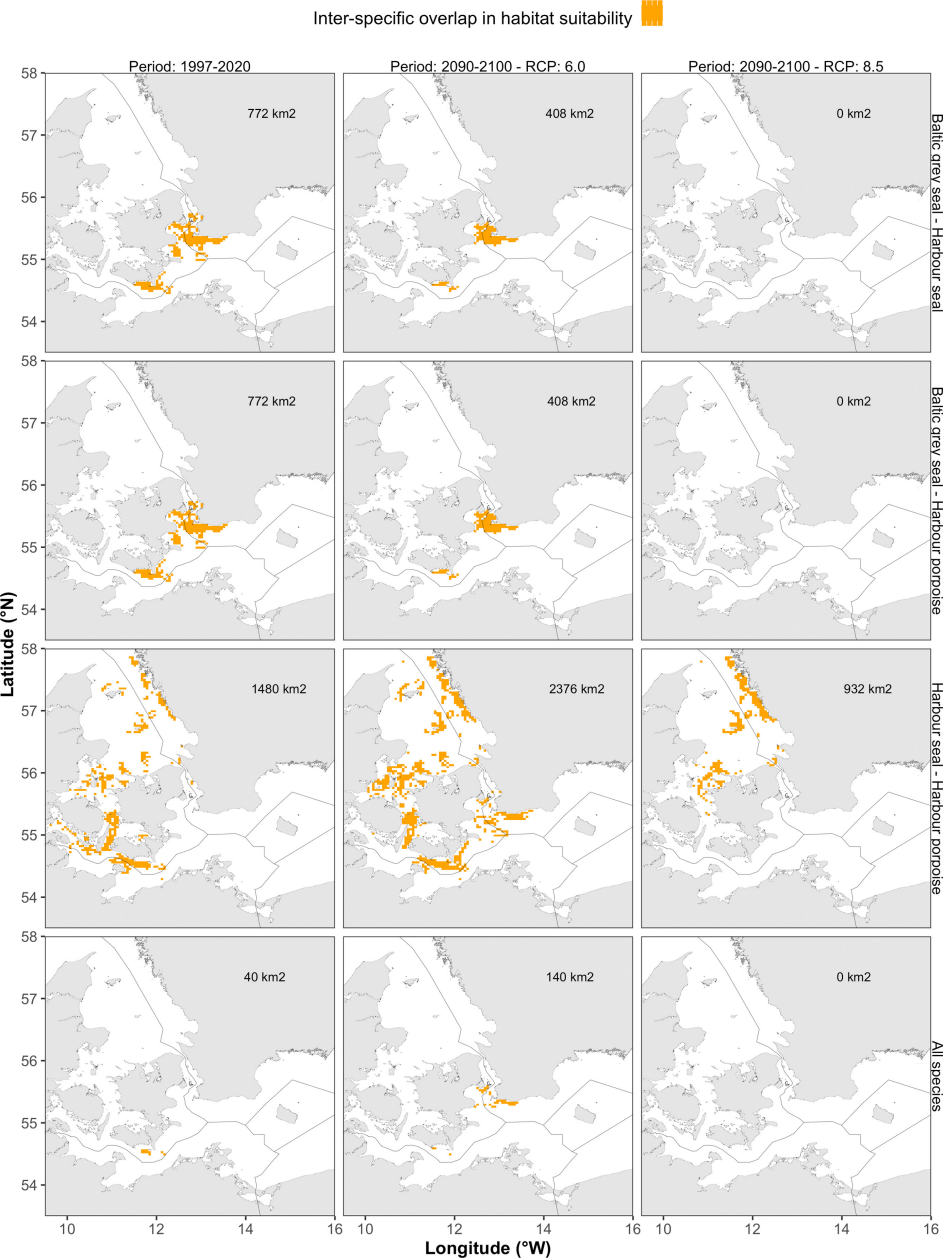
Maps of the inter‐specific overlap in highly suitable habitats (using the SDM threshold Kappa) for the periods 1997–2020 (current) and 2080–2100 (RCPs 6.0 and 8.5). All possible species combinations are shown with orange pixels indicating areas of expected overlap between species. The absolute area size of inter‐specific overlap in highly suitable habitats (km^2^) is provided in the top right corner of each panel

Inter‐specific overlap in areas predicted to contain highly suitable habitats (Kappa threshold) across all possible species combinations increased from 40 km^2^ to 140 km^2^ when comparing the total area size between the periods 1997–2020 and 2090–2100 for scenario RCP 6.0 (Figure 6). However, forecasting MaxEnt model results between the periods 1997–2020 and 2090–2100 for scenario RCP 8.5 suggested a complete loss of inter‐specific overlap in areas of highly suitable habitats for most species combinations. Here, only overlap in highly suitable habitats between harbor seals and harbor porpoises remained under scenario RCP 8.5, although overlap was predicted to decline to 932 km^2^ compared to 1480 km^2^ during the period 1997–2020 (Figure 6).

## 4 DISCUSSION

This study provides a comprehensive overview of potential changes between contemporary and future habitat suitability of three marine predators (Baltic gray seals, harbor seals, and harbor porpoises) and the implications for inter‐specific overlap within the southwestern Baltic Sea. Based on SDM predictions and IPCC‐based RCP scenarios, we show how divergent species‐specific responses to oceanographic variables may lead to spatial shifts and reduced availability of highly suitable habitats under future climatic conditions. Specifically, habitat suitability of harbor porpoises was predicted to increase slightly over time and space, while warmer and saltier waters and expected sea‐level rise under future climate reduced habitat suitability of harbor seals, especially of Baltic gray seals. Combined, the predicted geographic shifts under the most severe scenario RCP 8.5 may lead to a complete loss of spatial overlap between species in highly suitable habitats.

The loss of inter‐specific overlap in space under future conditions as predicted by our modeling approach was largely driven by a marked redistribution of area used by the predator guild under investigation. The forecasted reduction in highly suitable habitats available to Baltic gray seals under future conditions was a major contributor to the loss of inter‐specific overlap. Underlying this pattern was a negative effect of sea surface salinity on habitat suitability. The effect of sea surface salinity should, however, be interpreted with care as Baltic gray seals are currently recolonizing areas with relatively high salinity levels, such as the Kattegat in the northern part of our study area (Galatius et al., 2020). Unfortunately, location data of Baltic gray seals from this area are scant and most of the data used here were collected from individuals that use the brackish waters of the Baltic Sea and may, therefore, be more sensitive to an increasing salinity gradient than individuals in the northern part of the study area. Archeological data indicate that the gray seal was the most common seal species in the inner Danish waters including Kattegat from the 16^th^ to 19^th^ centuries before they were locally extinct around 1900 (Olsen et al., 2018). Genetic analyses of specimens from Kattegat from that time have shown all investigated gray seals from Kattegat to be of Baltic origin (Fietz et al., 2016). The historical and current presence of Baltic gray seals in Kattegat may indicate that the response of this species to salinity as estimated in our models may be an artifact of other factors co‐varying with salinity that are mostly relevant in the southern Baltic Sea.

Distance to haulout sites was another important predictor variable in the habitat suitability models of both seal species. This was to be expected given that seals need haulout sites to rest, molt, breed, and take care of their pups and thus frequently return to their preferred haulout sites (Sjöberg and Ball, 2000). As the climate warms and sea levels rise, some of the important haulout sites in the southwestern Baltic Sea and adjacent waters are expected to be flooded, and thus become unavailable (Figure S2.3 in Appendix S2). Loss of currently existing haulout sites following expected sea‐level rise in our study area was the main contributor to the predicted spatiotemporal decline in seal habitat suitability and subsequently inter‐specific overlap. Important to note is that in our models and forecasts, we did not allow new haulout sites to emerge as it is difficult to predict if and where new haulout sites will be established under future conditions. It is certainly possible that Baltic gray seals and harbor seals will begin to use new areas along the coastline as alternative haulout sites under future conditions, as has also been observed for ringed seals (*Pusa hispida*, Schreber, 1775) that are already under climate pressure (Lydersen et al., 2017). However, annual seal monitoring programs in the study area have not detected the establishment of new haulout sites over the last 20 years and we suspect that seals that potentially lose their preferred haulout site in the future are more likely to start using already existing haulout sites nearby, leading to increased local densities and lower occurrence in areas with large distances to the remaining haulouts. Thus, our findings may serve as an early warning signal that currently available haulout sites for seals in the southwestern Baltic Sea and adjacent waters are threatened by climate change. Future studies should try to identify areas along the Baltic Sea coastline where new haulout sites could potentially be established to inform marine species conservation initiatives and improve projections of future habitat suitability.

Habitat suitability of harbor porpoises was largely determined by variation in sea surface salinity, temperature, and seabed slope (i.e., variables with the highest model contribution or permutation importance). The importance of sea surface salinity aligns well with previous findings from the first MaxEnt model developed for this species from the same area (Edrén et al., 2010). Despite differences in temporal scale, model pruning, and development, Edrén et al. (2010) and our study show how habitat suitability of harbor porpoises tends to peak at intermediate salinity levels and tapers off at low and high salinity levels. These similarities in study results strengthen confidence in the reliability of our harbor porpoise habitat suitability maps under contemporary and future climate conditions.

Systematically collecting long‐term and precise location data of multiple marine predator species is challenging and expensive, and thus rare (Reisinger et al., 2022). The here analyzed location dataset is the most extensive that currently exists in the Baltic Sea region. Nonetheless, some challenges in the dataset required methodological consideration so as to reduce prediction uncertainty, which is often neglected in large‐scale SDM studies that consider possible climate‐change impacts (Beale and Lennon, 2012). For example, an important assumption of SDM studies is that sampling of location data is adequate and representative. We have already stated above that the location data of Baltic gray seals from this area are likely biased to the southern part of the study area. But in an attempt to fulfill this assumption as well as possible, we incorporated spatial sampling bias files in the species‐specific MaxEnt models, which is an established method to restrict background points to areas where species occurrences were found, leading to more realistic predictions (Phillips et al., 2009). We also tailored the entire analytical procedure to increase the reliability of model predictions by, e.g., excluding areas with novel environmental conditions, and limiting overparameterization through extensive MaxEnt model pruning (Kass et al., 2021). It is also important to highlight that the future distribution and habitat suitability of marine mammals is not only influenced by climate‐induced changes in oceanographic features such as sea‐level rise, surface temperature, and salinity. For example, anthropogenic activities such as commercial fisheries, chemical pollution, offshore wind farm construction, and shipping also occur widely throughout the Baltic Sea (Reusch et al., 2018) and may have marked effects on the current and future habitat suitability of marine predators through competition for fish (Hansson et al., 2018), wildlife health (Sonne et al., 2020), and disturbance through underwater noise (Jalkanen et al., 2018). However, it is currently unknown how, e.g., underwater noise, commercial fishing effort, and prey distribution will change under future conditions, and as such these candidate predictor variables were not considered in our study. This does not imply, however, that these variables do not affect the ecology of our study species and we recommend that future studies try to estimate their impacts on the habitat suitability of marine predators through, e.g., scenario‐based simulation models. Despite these caveats, our results clearly indicate that ongoing climate warming is likely to have a strong impact on marine predators in the southwestern part of the Baltic Sea, including the Danish straits and Kattegat, with directional shifts in species' habitat suitability and overlap. To what extent the observed changes in inter‐specific overlap of habitat suitability under future conditions will alter inter‐specific competition, local food‐web dynamics, and possibly ecosystem functioning (Doney et al., 2012) remain important questions for future research.

## AUTHOR CONTRIBUTIONS

**Floris Michiel van Beest:** Conceptualization (lead); data curation (lead); formal analysis (lead); methodology (lead); visualization (lead); writing – original draft (lead). **Rune Dietz:** Data curation (equal); writing – review and editing (equal). **Anders Galatius:** Data curation (equal); writing – review and editing (equal). **Line A. Kyhn:** Data curation (equal); writing – review and editing (equal). **Signe Sveegaard:** Data curation (equal); writing – review and editing (equal). **Jonas Teilmann:** Conceptualization (equal); data curation (equal); writing – review and editing (equal).

## CONFLICT OF INTEREST

The authors declare that they have no known competing financial interests or personal relationships that could have appeared to influence the work reported in this article.

## Supporting information


Appendix S1

Appendix S2

## Data Availability

Data used in this study is available through the repository Dryad: https://datadryad.org/stash/share/HWuETLTr‐0Hc8Rq8pqU_0kfWcth6jYD8qV3QXZgAtZk.
