# Supplementary material for: Forecasting shifts in habitat suitability of three marine predators suggests a rapid decline in inter‐specific overlap under future climate change
Source: Ecol Evol. 2022 Jul 6;12(7):e9083. doi: 10.1002/ece3.9083 (PMC9257519; doi:10.1002/ece3.9083)
Supplement: Supplementary file 1 — Appendix S1 [file ECE3-12-e9083-s001.docx]

**Appendix S1**.

Overview, Data, Model, Assessment and Prediction (ODMAP) protocol (*sensu* Zurell et al., 2020).

| ODMAP element | Contents |
| --- | --- |
| **OVERVIEW** |  |
| *Authorship* | - **Authors**: Floris M. van Beest, Rune Dietz, Anders Galatius, Line Anker Kyhn, Signe Sveegaard, Jonas Teilmann - **Contact email**: flbe@ecos.au.dk - **Title**: Forecasting shifts in habitat suitability of three sympatric marine predators suggests a rapid decline in inter-specific overlap under future climate change |
| *Model objective* | - **Objective**: To predict spatiotemporal changes in habitat suitability and overlap among three marine predators co-occurring in the southwestern Baltic Sea including the Kattegat. - **Target outputs**: Species-specific maps of contemporary (1997-2020) and future (2090-2100) habitat suitability and inter-specific overlap. |
| *Taxon* | - Baltic grey seal (*Halichoerus grypus grypus*) - Harbour seal (*Phoca vitulina*) - Harbour porpoise (*Phocoena phocoena*) |
| *Location* | Southwestern part of Baltic Sea including the Danish Straights and the Kattegat (ca. 156.000 km^2^) see Figure 1 in the main article. |
| *Scale of analysis* | - **Spatial extent**: Longitude 9°E-16°E, Latitude 53.5°N-58°N - **Spatial resolution**: 9.2 km^2^ - **Temporal extent/time period**: contemporary: 1997-2020, future: 2090-2100. - **Type of extent boundary**: administrative (southwestern Baltic Sea and the Kattegat) following the Helsinki Convention on the Protection of the Marine Environment of the Baltic Sea Area. |
| *Biodiversity data overview* | - **Observation type**: Tracking (location) data collected through animal-borne ARGOS- and GPS-tags. - **Response/Data type**: Presence-only |
| *Type of predictors* | A total of 7 predictor variables were considered (see section predictor variables under DATA element for further details), reflecting gradients in environmental conditions (i.e. bathymetry, sea bed slope, distance to nearest haulout, sediment type, sea surface current velocity), climatic conditions (i.e. sea surface salinity, sea surface temperature) within the study area. All predictor data were downloaded or self-generated as raster maps (Table 1 in the main article). |
| *Conceptual model / Hypothesis* | **A conceptual diagram** of the habitat suitability modelling procedure is provided in Figure S1.1 below. Presence-only data of Baltic grey seal, harbour seal and harbour porpoises were matched with predictor variables aggregated over the period 1997-2020 within a machine learning (MaxEnt) model framework to estimate habitat suitability under contemporary conditions. Future habitat suitability was predicted through model forecasting using the same predictor variables aggregated over the period 2090-2100. Shifts and changes in habitat suitability between periods were quantified for each species, each Representative Concentration Pathway (RCP) scenario (based on the mean of 3 Global Circulation Models (GCMs)) and 3 habitat suitability thresholds (Kappa, MSSS and P10) separately and subsequently used to assess changes in inter-specific overlap.  **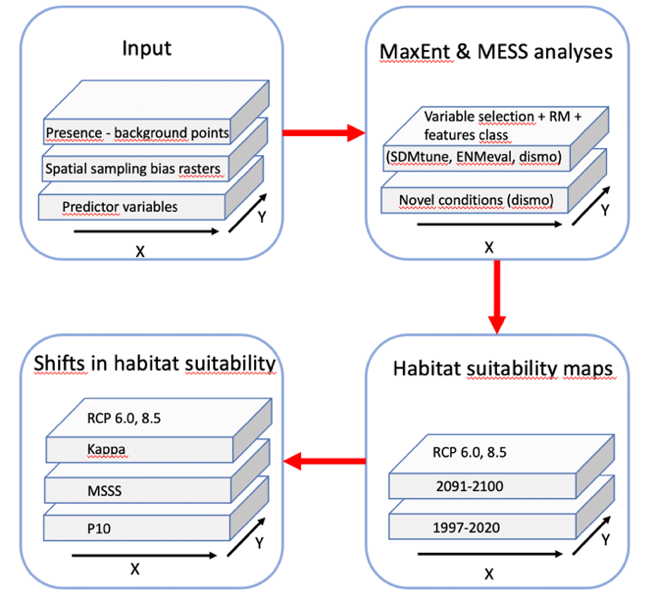 Figure S1.1:** *Conceptual diagram of the analytical procedure showing the input data and MaxEnt analyses used to construct habitat suitability maps and quantify shifts over time and space based on various RCP scenarios. Novel conditions in the future were identified and excluded from model predictions using MESS analysis (see Prediction section below). R-packages used in the analyses are provided in brackets* (dismo: Hijmans et al., 2017; ENMeval: Kass et al., 2021; SDMtune: Vignali et al., 2020)*.*  **Hypotheses**: We expected forecasted changes in climatic sensitive oceanographic conditions (i.e. sea surface salinity, sea surface temperature) to alter future habitat suitability compared to the present situation. If these co-occuring species respond differently to such future conditions, we expected altered habitat suitability to lead to a redistribution of area use while at sea and consequently change inter-specific overlap. |
| *Assumptions* | This study is based on three simplifying assumptions typical for species distribution models constructed with presence-only data (Radosavljevic & Anderson, 2014):   - Sampling of location data was adequate and representative (see “Scaling and bias” section under “biodiversity data” for the approach incorporated to minimize the effects of bias in sampling of locations). - Detectability was constant across the environment. - Species were at equilibrium with their environment. |
| *SDM algorithms* | - **Algorithm**: MaxEnt was chosen due to its competitive performance for small sample sizes and presence-only data (Elith et al., 2006; Wisz et al., 2008). - **Model complexity**: We used specifies-specific model pruning to restrict complexity while retaining ecological realism and optimizing predictive performance (see section on model settings below for details). |
| *Model workflow* | Model structure and complexity were pruned for each species separately to optimize predictive performance and minimize overfitting (using 80% for model training (calibration) and 20% for model testing (evaluation)). Optimal model settings were selected based on two sequential criteria. First, we filtered candidate models with OR10 <10% (to avoid variable overfitting) and then selecting the model with the highest predictive performance as determined by the area under the receiver operating characteristic curve (AUC) value. The ‘optimal model’ was used to predict species habitat suitability maps (i.e. probability of occurrence ranging from 0 to 1) for each period based on the mean cloglog output of 10 replicate model runs. Predictions under future conditions were made using the RCP scenarios 6.0 and 8.5. |
| *Software* | - **Software**: R version 4.0.3 (2020-10-10), MaxEnt version 3.4.1 - **Data availability**: Data used in this study is available through the repository Dryad: <https://datadryad.org/stash/share/HWuETLTr-0Hc8Rq8pqU_0kfWcth6jYD8qV3QXZgAtZk>. |
| **DATA** |  |
| *Biodiversity data* | - **Taxon names**: Baltic grey seal (*Halichoerus grypus grypus*), Harbour seal (*Phoca vitulina*), Harbour porpoise (*Phocoena phocoena*). - **Taxonomic reference system**: Integrated Taxonomic Information System (ITIS) - **Ecological level**: Species level and landscape-scale - **Data sources:** - Location data: collected by fitting individuals of each species with Argos-tags or GPS-tags (Figure 2 in main article). - **Sampling design**: Individual seals tracked during this study were actively captured on haulout sites in Denmark and Sweden. Individual porpoises were caught incidentally in pound nets, which are used in near-shore commercial fisheries in the inner Danish waters. Each individual animal was subsequently fitted with either an Argos-tag or GPS-tag to collect location data. Argos tags-were programmed to make a limited number of satellite uplinks and acquire a location at pre-defined times (duty cycles) to increase battery life. Duty cycles varied between 1 and 4 d. The GPS-tags attempted to acquire and store a location every 3rd min (porpoise tags) or during each surfacing attempt (seals). - **Sample size:** Location data (Figure 2 in the main article) was collected for 31 Baltic grey seals (13 Argos and 18 GPS), 74 harbour seals (57 Argos and 17 GPS), and 132 harbour porpoises (123 Argos and 9 GPS). - **Mask:** All presence and background locations as well as predictor variable data were restricted to the marine environment, effectively removing all location and raster data with land from the analyses. - **Scaling and bias:** Spatial sampling bias files were constructed for each species separately by computing Gaussian kernel density raster of all sampling locations (Figure S2.4 in Appendix S2). Bias rasters were subsequently included in the MaxEnt models to up‐weight presence‐only data points with fewer neighbours in the geographic landscape and, moreover, to restrict sampling of background points to areas where occurrences were found (Phillips et al., 2009). - **Data filtering:** First, location data collected within 24 hours after tagging were discarded to reduce behavioural bias caused by capture and tagging. Second, locations on land were removed from the data. Third, location data were screened for impossible movements based on swimming speed between consecutive locations and if detected one of the two locations was removed from the data (for details see: Sveegaard et al., 2011; van Beest et al., 2018, 2019). Finally, the GPS location data was subsampled to reduce autocorrelation. To this end we only used locations collected as close as possible to the hours 03:00, 9:00, 15:00, and 21:00. - **Absence data**: No true absence data were available. |
| *Data partitioning* | Throughout the analytical procedure, species-specific location data were partitioned using 80% for model training (calibration) and 20% for model testing (evaluation). We randomly drew one set of background locations for each species separately, while accounting for spatial sampling bias (see section Biodiversity data – scaling and bias above). We always applied masked geographically structured data partitioning through the ‘block’ method in the ‘ENMeval’ package in R (Muscarella et al., 2014). With this method occurrence locations are partitioned into four bins depending on their geographic position. In each iteration, models were trained using k-1 bins and evaluated on the withheld bin. Background localities are restricted to bins corresponding to the occurrence data. Such ‘masked geographically structured’ data partitioning approaches are expected to yield best results in model selection and more honest predictions compared to split and unmasked spatial partitioning (Radosavljevic & Anderson, 2014; Santini et al., 2021). |
| *Environmental data/ predictor variables* | - **Predictor variables:** The variables: “sea surface current velocity (m/s)”, “sea surface salinity (PSU)” and “sea surface temperature (°C)” were freely available and downloaded through the Bio-ORACLE (v2.2) database (https://bio-oracle.org/) at a 5 arcmin (9.2 km) resolution (Assis et al., 2018; Tyberghein et al., 2012). Bio-ORACLE variables represent averaged monthly values over the period 2000-2014 (current period) and 2091-2100 (future period). For projected future conditions, Bio-ORACLE averaged data produced by three global circulation models (GCMs) for four scenarios of representative concentration pathways (RCPs: 2.6, 4.5 6.0 and 8.5). Specifically, the three selected GCMs are part of the CMIP5 collection of model runs used in IPCC’s 5th Assessment Report (IPCC, 2013) and were CCSM4 (Drake et al., 2016), HadGEM2-ES (Jones et al., 2011), and MIROC5 (Watanabe et al., 2010). Here we chose to consider only RCP scenarios 6.0 and 8.5 as these are the most likely future states given current emission rates (Schwalm et al., 2020). The RCP 6.0 scenario represents a high greenhouse gas emission scenario in which total radiative forcing is stabilized after 2100 with global mean temperatures projected to rise by about 2.2°C in year 2100. RCP 8.5 represents a severe emission scenario, with emissions following the same trajectory as during the last decade with global temperatures expected to increase by about 4°C in year 2100 relative to 1850–1900.   The variables “bathymetry (m)” and “sea bed slope (°)” were calculated based on a digital terrain model (500 m resolution) of the region freely available and downloaded through the HELCOM database (https://maps.helcom.fi/). The variable “sediment type” is a categorical variable including sand, clay, mud, bedrock and hard bottom complex, was extracted from a raster file (300 m resolution) accessed through the Baltic Sea Management – Nature Conservation and Sustainable Development of the Ecosystem through HELCOM (http://www.helcom.fi/baltic-sea-trends). Distance to nearest haulout was calculated as the Euclidian distance (km) between each seal location and the closest known haulout site in the region. Seal haulout data were obtained from <https://sharkweb.smhi.se/hamta-data/> (Sweden), Aarhus University seal database (Denmark) and German Oceanographic Museum database (Germany). All raster data were resampled to 9.2 km using bilinear interpolation to match Bio-ORACLE data. While bathymetry, sea bed slope and sediment type were static variables, distance to nearest haulout was recalculated under future climate change conditions by considering a global mean sea level rise, resulting from ice melt and steric rise, of 0.39 m and 0.65 m for RCP scenarios 6.0 and 8.5 respectively (Grinsted, 2015; Katsman et al., 2011; Marzeion et al., 2012). Depending on the haulout location, isostatic water level rises of 0.10 m in the southwestern Baltic Sea, -0.05 m in southern Kattegat and around Bornholm, and -0.15 m in central and northern Kattegat (Grinsted, 2015; Rosentau et al., 2012) were added to the mean sea level rise. Accurate elevation data for seal haulout sites in this area are not known, but were based on judgement by two authors (AG and JT) who are familiar with these haulout sites. The water level rises effectively removed some currently available haulout sites from future use  See Figure S2.2 in Appendix S2 for an overview of raster layers of predictor variables in the southwestern Baltic Sea and Kattegat as included in the MaxEnt models and Figure S2.3 in Appendix S2 for an overview of haulout locations.   - **Data sources:** Candidate predictor variables (Table 1 in the main article) consisted of variables freely available through the Bio-ORACLE (v2.2) database (<https://bio-oracle.org/>), the HELCOM database (<https://maps.helcom.fi/>) and Sharkweb (<https://sharkweb.smhi.se/>) - **Spatial resolution and extent of raw data:** All raster variables were clipped to the study area extent: Longitude 9°E-16°E, Latitude 53.5°N-58°N and resampled to a spatial resolution (grid cell size) of 9.2km^2^. - **Temporal resolution and extent of raw data:** The Bio-ORACLE variables represent averaged monthly values over the period 2000-2014 (current period) and 2091-2100 (future period). The variable distance to nearest known haulout site was calculated for the period 1997-2020 using haulout sites surveyed annually (Table 1 in main article) and recalculated for the period 2091-2100 using global mean sea level rise values and local isostatic water level rises as described above. - **Geographic projection:** WGS 1984 (coordinate reference system in R: “+proj=longlat +datum=WGS84 +no_defs +ellps=WGS84 +towgs84=0,0,0) - **Data processing:** Data processing was limited to clipping raster data to the study area extent, and resampling data to a 9.2 km resolution using the “aggregate” function from the ‘raster’ package in R. |
| **MODEL** |  |
| *Variable pre-selection* | No variable pre-selection was considered. |
| *Multicollinearity* | Multicollinearity was assessed by calculating the variance inflation factor (VIF) among the 7 predictor variables using the “usdm” package in R (Naimi et al., 2014). The results revealed all VIF values to be <3, which suggests that multicollinearity was not of great concern in our data (Dormann et al., 2013). |
| *Model settings* | - **Background points**: MaxEnt relies on background points to characterize the environmental conditions within the area of interest. Prior to generating background points, we generated spatial sampling bias files for each species separately by computing Gaussian kernel density rasters of all sampling locations. Bias raster files were subsequently included in the species-specific MaxEnt models to up‐weight presence‐only data points with fewer neighbours in the geographic landscape and to restrict background points to geographic areas where species occurrences were found (Phillips et al., 2009). Thus, while accounting for spatial sampling bias, we randomly drew one set of background locations (10 000) for each species separately. - **Variable selection**: No variable selection was used as multicollinearity was low and we preferred to consider all covariates in the species-specific models to facilitate comparison of variable importance. - **Model pruning and selection of settings:** MaxEnt uses regularization multipliers (RM) to protect against overfitting and to reduce model complexity (Phillips et al., 2006). RMs give a penalty for each term included in the model and for higher weights given to a term. Here, we tested different settings of RM using the range of 0.5-5.0 in increments of 0.5 for each feature class through the ‘ENMeval’ package in R (Muscarella et al., 2014). We restricted each feature class to ‘linear’, ‘quadratic’ or ‘ linear and quadratic’ functions to avoid overly complex and implausible response curves that would be hard to explain ecologically. The amount of overfitting for each candidate model was subsequently quantified by calculating the ‘10% training omission rate’ (OR10). OR10 values greater than 10% typically indicate model overfitting. From the set of candidate models, we selected the optimal model settings (i.e. RM and feature class) using two sequential criteria. First, we filtered candidate models with OR10 <10% and then selecting the model with the highest AUC (Kass et al., 2021). |
| *Model estimates* | MaxEnt generates two measures of predictor variable importance to describe the relative contribution of predictor variables to a model (Figure S2.6 in Appendix S2). Percent contribution expresses the amount of explained variance each variable contributes, while permutation importance measures how AUC values change when a variable is removed from the model, with a large decrease indicating a strong dependence of the model on that variable. The values are normalized to give percentages. |
| *Model averaging / Ensembles* | No ensemble modelling was considered here as sample sizes were relatively low for some species and MaxEnt is known to perform well under such conditions. Model averaging was done by fitting the model with the optimal settings 10 times using different test and training datasets for each run. This allowed us to extract habitat suitability thresholds, AUC values, variable importance and response data for each model run and assess model uncertainty. |
| *Non-independence* | Non-independence of location data across the latitudinal gradient was tackled by sub-sampling location data as explained above, which reduced autocorrelation to acceptable levels (Figure S2.1 in Appendix S2). |
| *Threshold selection* | To quantify shifts in habitat suitability between periods, three thresholds for the probability of occurrence were used: i) Kappa (the value of the probability of occurrence at which Kappa is highest), ii) MSSS (the value of the probability of occurrence at which the sum of the sensitivity (true positive rate) and specificity (true negative rate) is maximized), and iii) P10 (the value of the probability of occurrence for the lowest 10% of occurrence records). Species specific threshold values are provided in Table S2.2 in Appendix S2. In general, the Kappa threshold is the most restrictive as it serves to identify habitat with relatively high suitability. The MSSS threshold identifies habitat above a moderate suitability, while the P10 threshold includes most habitat above a relatively low suitability. |
| **ASSESSMENT** |  |
| *Performance statistics* | Predictive accuracy of the species-specific models was assessed through the AUC value, where models with AUC values above 0.7 are usually considered useful (Elith et al., 2006). |
| *Plausibility checks* | Response curves (i.e. partial dependence plots) were plotted to assess the ecological plausibility of the relationship between each predictor variable and predicted habitat suitability (Figure S2.7 in Appendix S2). |
| **PREDICTION** |  |
| *Prediction output* | All habitat suitability maps are based on the complimentary log-log (cloglog) output of MaxEnt (default in version 3.4.1 (Phillips et al., 2017)), which can be interpreted as a spatially-explicit probability of habitat suitability (ranging from 0 to 1), conditioned on the predictor variables included in the models.  Prior to predicting habitat suitability under current and future climatic conditions, a multivariate environmental similarity surfaces (MESS) analysis was performed to identify areas with novel environmental conditions (i.e. conditions the model has no data on, rendering predictions unreliable). Following Elith et al., (2010), the presence locations under current conditions (1997-2020) were provided as input points alongside raster data of current and future conditions. Here we considered only the variables sea surface temperature and salinity, current velocity and distance to closest haulout (for the seal species) as these were the only variables expected to change under future climatic conditions. The MESS analysis then estimated (dis)similarities in environmental conditions used and available across the study area extent for each period. The MESS analysis was done for each species and RCP scenario seperately. Based on the MESS output, we only retained those areas where environmental conditions were similar over time (Figure S2.5 in Appendix S2). In other words, we removed (i.e. masked) all areas with conditions that ranged outside those conditions currently used or found (negative MESS values) prior to predicting habitat suitability over time and space. |
| *Uncertainty quantification* | There are numerous sources of uncertainty when predicting habitat suitability over time and space based on presence only data and climate change models. Therefore, we tailored the entire analytical procedure to minimize uncertainty in model output as much as possible by e.g. incorporating spatial sampling bias files, limiting overparameterization, and using spatial block validation in the MaxEnt model pruning (Santini et al., 2021). Our analyses also considered three GCMs for each RCP scenario, which is a known source of uncertainty in climate change studies (Thuiller et al., 2019). |

**References**

Assis, J., Tyberghein, L., Bosch, S., Verbruggen, H., Serrão, E. A., & Clerck, O. De. (2018). Bio-ORACLE v2.0: Extending marine data layers for bioclimatic modelling. *Global Ecology and Biogeography*, *27*(3), 277–284. https://doi.org/10.1111/GEB.12693

Dormann, C. F., Elith, J., Bacher, S., Buchmann, C., Carl, G., Carré, G., Marquéz, J. R. G., Gruber, B., Lafourcade, B., Leitão, P. J., Münkemüller, T., McClean, C., Osborne, P. E., Reineking, B., Schröder, B., Skidmore, A. K., Zurell, D., & Lautenbach, S. (2013). Collinearity: a review of methods to deal with it and a simulation study evaluating their performance. *Ecography*, *36*(1), 27–46. https://doi.org/10.1111/J.1600-0587.2012.07348.X

Drake, J. B., Jones, P. W., & George R. Carr, J. (2016). Overview of the Software Design of the Community Climate System Model: *Http://Dx.Doi.Org/10.1177/1094342005056094*, *19*(3), 177–186. https://doi.org/10.1177/1094342005056094

Elith, J., H. Graham, C., P. Anderson, R., Dudík, M., Ferrier, S., Guisan, A., J. Hijmans, R., Huettmann, F., R. Leathwick, J., Lehmann, A., Li, J., G. Lohmann, L., A. Loiselle, B., Manion, G., Moritz, C., Nakamura, M., Nakazawa, Y., McC. M. Overton, J., Townsend Peterson, A., … E. Zimmermann, N. (2006). Novel methods improve prediction of species’ distributions from occurrence data. *Ecography*, *29*(2), 129–151. https://doi.org/10.1111/j.2006.0906-7590.04596.x

Elith, J., Kearney, M., & Phillips, S. (2010). The art of modelling range-shifting species. *Methods in Ecology and Evolution*, *1*(4), 330–342. https://doi.org/10.1111/j.2041-210X.2010.00036.x

Hijmans, R. J., J. Phillips, S., & Elith, J. (2017). *dismo: Species Distribution Modeling*. https://CRAN.R-project.org/package=dismo.

IPCC. (2013). Climate change 2013 - The Physical Science Basis Contribution of Working Group I to the Fifth Assessment Report of the Intergovernmental Panel on Climate Change. In T. Stocker, Q. Dahe, G.-K. Plattner, M. Tignor, A. Sk, B. J, N. A, Y. Xia, V. Bex, & M. PM (Eds.), *Contribution of Working Group I to the Fourth Assessment Report of the Intergovernmental Panel on Climate Change*. Cambridge University Press.

Jones, C. D., Hughes, J. K., Bellouin, N., Hardiman, S. C., Jones, G. S., Knight, J., Liddicoat, S., O’Connor, F. M., Andres, R. J., Bell, C., Boo, K. O., Bozzo, A., Butchart, N., Cadule, P., Corbin, K. D., Doutriaux-Boucher, M., Friedlingstein, P., Gornall, J., Gray, L., … Zerroukat, M. (2011). The HadGEM2-ES implementation of CMIP5 centennial simulations. *Geoscientific Model Development*, *4*(3), 543–570. https://doi.org/10.5194/GMD-4-543-2011

Kass, J. M., Muscarella, R., Galante, P. J., Bohl, C. L., Pinilla-Buitrago, G. E., Boria, R. A., Soley-Guardia, M., & Anderson, R. P. (2021). ENMeval 2.0: Redesigned for customizable and reproducible modeling of species’ niches and distributions. *Methods in Ecology and Evolution*, *00*, 1–7. https://doi.org/10.1111/2041-210X.13628

Muscarella, R., Galante, P. J., Soley-Guardia, M., Boria, R. A., Kass, J. M., Uriarte, M., & Anderson, R. P. (2014). ENMeval: An R package for conducting spatially independent evaluations and estimating optimal model complexity for Maxent ecological niche models . *Methods in Ecology and Evolution*, *5*(11), 1198–1205. https://doi.org/10.1111/2041-210x.12261

Naimi, B., Hamm, N. A. S. S., Groen, T. A., Skidmore, A. K., & Toxopeus, A. G. (2014). Where is positional uncertainty a problem for species distribution modelling? *Ecography*, *37*(2), 191–203. https://doi.org/10.1111/j.1600-0587.2013.00205.x

Phillips, S. J., Anderson, R. P., Dudík, M., Schapire, R. E., & Blair, M. E. (2017). Opening the black box: an open-source release of Maxent. *Ecography*, *40*(7), 887–893. https://doi.org/10.1111/ecog.03049

Phillips, S. J., Anderson, R. P., & Schapire, R. E. (2006). Maximum entropy modeling of species geographic distributions. *Ecological Modelling*, *190*(3), 231–259. https://doi.org/https://doi.org/10.1016/j.ecolmodel.2005.03.026

Phillips, S. J., Dudík, M., Elith, J., Graham, C. H., Lehmann, A., Leathwick, J., & Ferrier, S. (2009). Sample selection bias and presence-only distribution models: Implications for background and pseudo-absence data. *Ecological Applications*, *19*(1), 181–197. https://doi.org/10.1890/07-2153.1

Radosavljevic, A., & Anderson, R. P. (2014). Making better Maxent models of species distributions: Complexity, overfitting and evaluation. *Journal of Biogeography*, *41*(4), 629–643. https://doi.org/10.1111/jbi.12227

Santini, L., Benítez-López, A., Čengić, M., Maiorano, L., & Huijbregts, M. A. J. (2021). Assessing the reliability of species distribution projections in climate change research. *Diversity and Distributions*, *00*, 1– 16. https://doi.org/10.1101/2020.06.10.143917

Schwalm, C. R., Glendon, S., & Duffy, P. B. (2020). RCP8.5 tracks cumulative CO2 emissions. *Proceedings of the National Academy of Sciences*, *117*(33), 19656–19657. https://doi.org/10.1073/PNAS.2007117117

Sveegaard, S., Teilmann, J., Tougaard, J., Dietz, R., Mouritsen, K. N., Desportes, G., & Siebert, U. (2011). High-density areas for harbor porpoises (*Phocoena phocoena*) identified by satellite tracking. *Marine Mammal Science*, *27*(1), 230–246. https://doi.org/10.1111/j.1748-7692.2010.00379.x

Thuiller, W., Guéguen, M., Renaud, J., Karger, D. N., & Zimmermann, N. E. (2019). Uncertainty in ensembles of global biodiversity scenarios. *Nature Communications*, *10*(1), 1–9. https://doi.org/10.1038/s41467-019-09519-w

Tyberghein, L., Verbruggen, H., Pauly, K., Troupin, C., Mineur, F., & Clerck, O. De. (2012). Bio-ORACLE: a global environmental dataset for marine species distribution modelling. *Global Ecology and Biogeography*, *21*(2), 272–281. https://doi.org/10.1111/J.1466-8238.2011.00656.X

van Beest, F. M., Mews, S., Elkenkamp, S., Schuhmann, P., Tsolak, D., Wobbe, T., Bartolino, V., Bastardie, F., Dietz, R., von Dorrien, C., Galatius, A., Karlsson, O., McConnell, B., Nabe-Nielsen, J., Olsen, M. T., Teilmann, J., & Langrock, R. (2019). Classifying grey seal behaviour in relation to environmental variability and commercial fishing activity - a multivariate hidden Markov model. *Scientific Reports*, *9*(1), 5642. https://doi.org/10.1038/s41598-019-42109-w

van Beest, F. M., Teilmann, J., Dietz, R., Galatius, A., Mikkelsen, L., Stalder, D., Sveegaard, S., & Nabe-Nielsen, J. (2018). Environmental drivers of harbour porpoise fine-scale movements. *Marine Biology*, *165*(5), 95. https://doi.org/10.1007/s00227-018-3346-7

Vignali, S., Barras, A. G., Arlettaz, R., & Braunisch, V. (2020). *SDMtune* : An R package to tune and evaluate species distribution models. *Ecology and Evolution*, ece3.6786. https://doi.org/10.1002/ece3.6786

Watanabe, M., Suzuki, T., O’Ishi, R., Komuro, Y., Watanabe, S., Emori, S., Takemura, T., Chikira, M., Ogura, T., Sekiguchi, M., Takata, K., Yamazaki, D., Yokohata, T., Nozawa, T., Hasumi, H., Tatebe, H., & Kimoto, M. (2010). Improved climate simulation by MIROC5: Mean states, variability, and climate sensitivity. *Journal of Climate*, *23*(23), 6312–6335. https://doi.org/10.1175/2010JCLI3679.1

Wisz, M. S., Hijmans, R. J., Li, J., Peterson, A. T., Graham, C. H., Guisan, A., Elith, J., Dudík, M., Ferrier, S., Huettmann, F., Leathwick, J. R., Lehmann, A., Lohmann, L., Loiselle, B. A., Manion, G., Moritz, C., Nakamura, M., Nakazawa, Y., Overton, J. M. C., … Zimmermann, N. E. (2008). Effects of sample size on the performance of species distribution models. *Diversity and Distributions*, *14*(5), 763–773. https://doi.org/10.1111/j.1472-4642.2008.00482.x

Zurell, D., Franklin, J., König, C., Bouchet, P. J., Dormann, C. F., Elith, J., Fandos, G., Feng, X., Guillera‐Arroita, G., Guisan, A., Lahoz‐Monfort, J. J., Leitão, P. J., Park, D. S., Peterson, A. T., Rapacciuolo, G., Schmatz, D. R., Schröder, B., Serra‐Diaz, J. M., Thuiller, W., … Merow, C. (2020). A standard protocol for reporting species distribution models. *Ecography*, ecog.04960. https://doi.org/10.1111/ecog.04960
