# Supplementary material for: Forecasting shifts in habitat suitability of three marine predators suggests a rapid decline in inter‐specific overlap under future climate change
Source: Ecol Evol. 2022 Jul 6;12(7):e9083. doi: 10.1002/ece3.9083 (PMC9257519; doi:10.1002/ece3.9083)
Supplement: Supplementary file 2 — Appendix S2 [file ECE3-12-e9083-s002.pdf]

Appendix S2.

Supplementary Figures and Tables

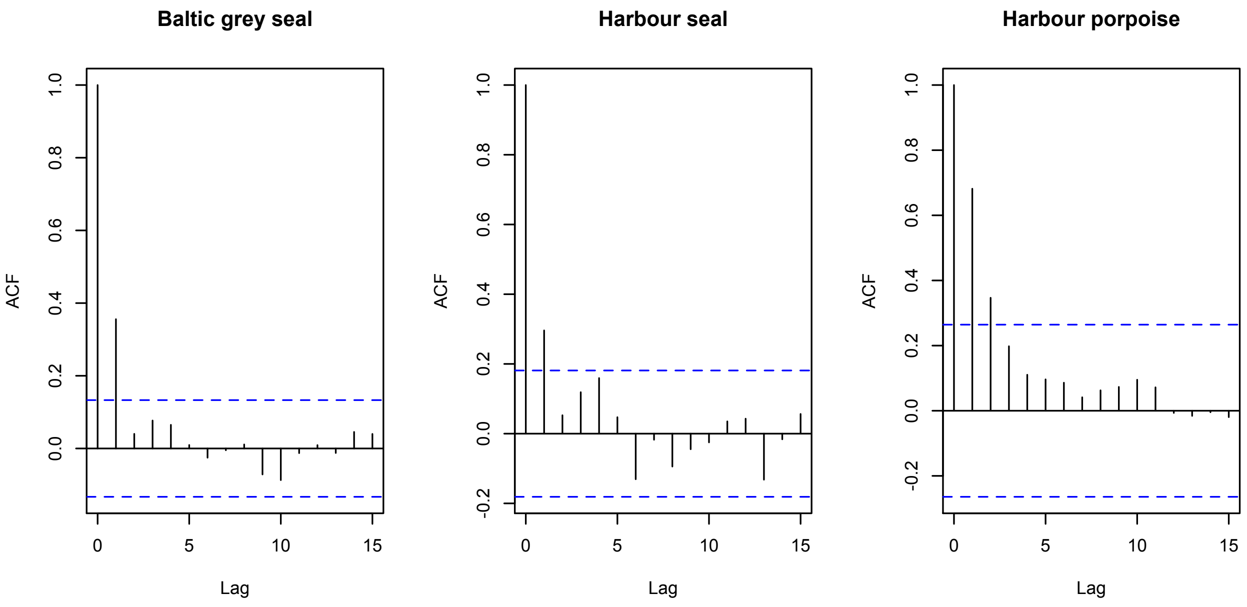

Figure S2.1: Species-specific autocorrelation function plots of the location data collected across the latitudinal gradient during 1997-2020 in the southwestern part of Baltic Sea, including the Danish Straits and the Kattegat. Lag units are 6-hour intervals due to subsampling of location data as described in the methods.

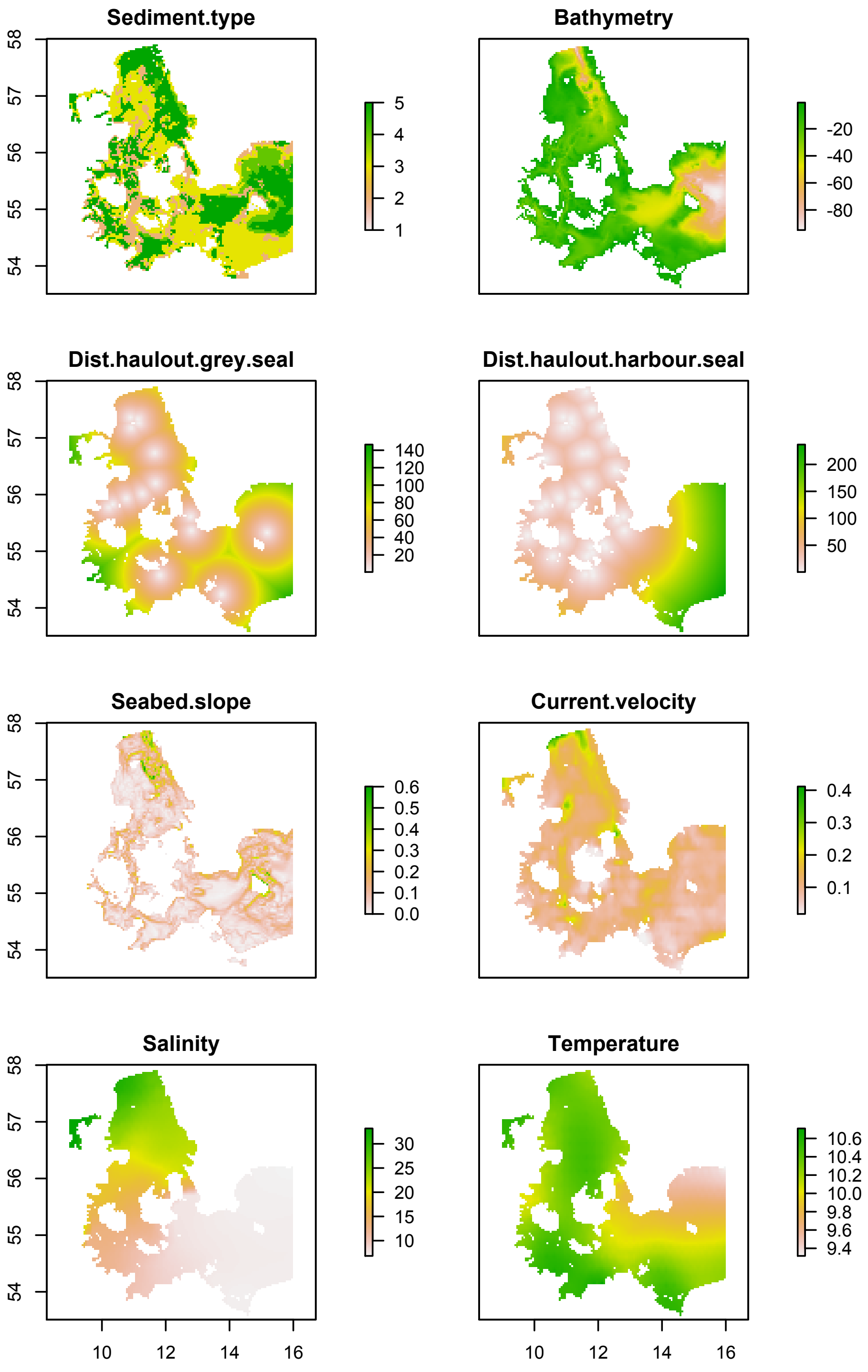

Figure S2.2: Raster layers of predictor variables in the southwestern Baltic Sea, including the Danish Straits and Kattegat as considered in the MaxEnt model for the period 1997-2020. See Table 1 in the main article for units and source of data.

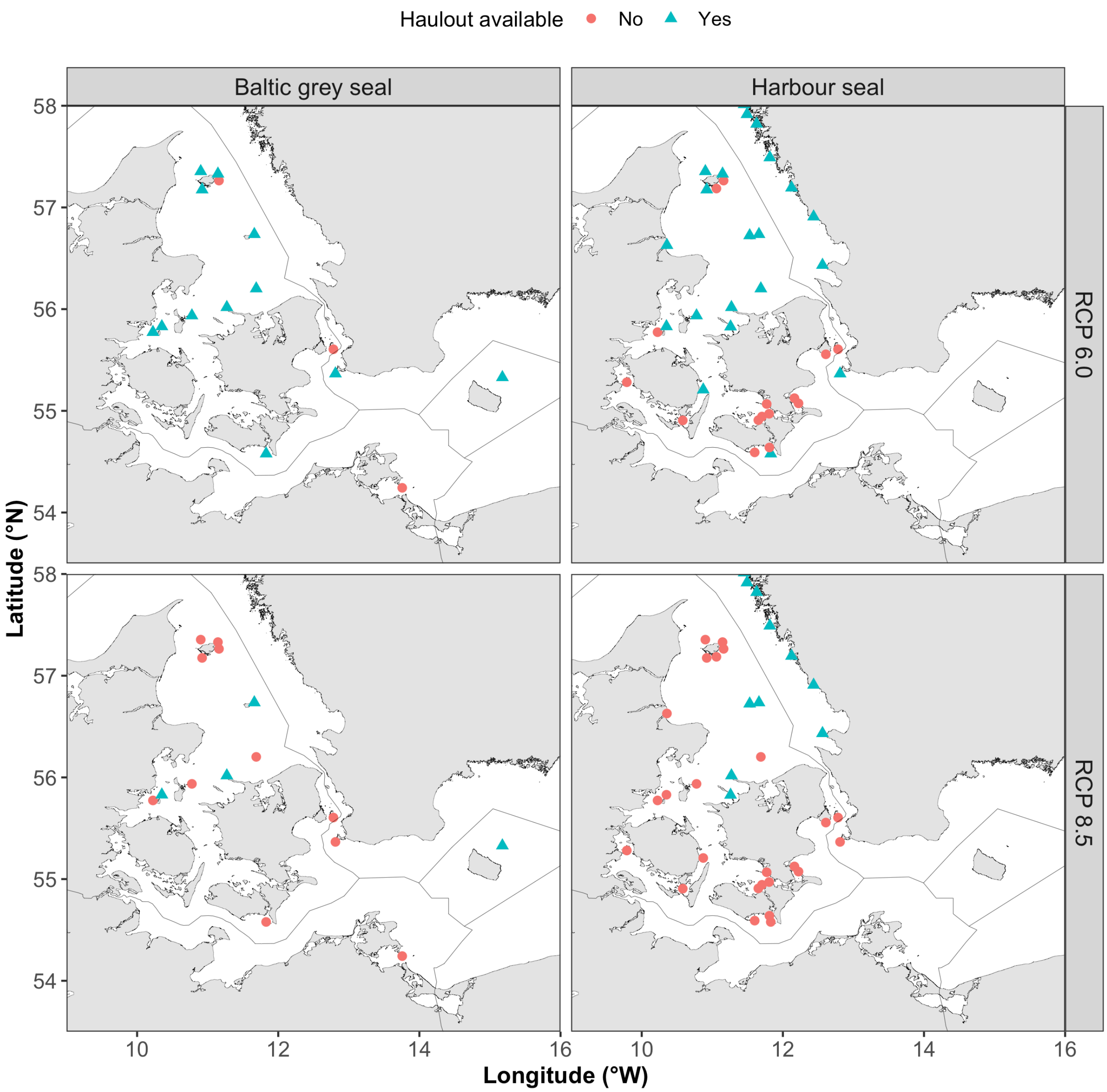

Figure S2.3 Map showing all known haulout sites for both Baltic grey seals and harbour seals within the study area including Danish, Swedish and German waters. Haulout sites are colour-coded based on their availability in the future following expected sea level rise within the area.

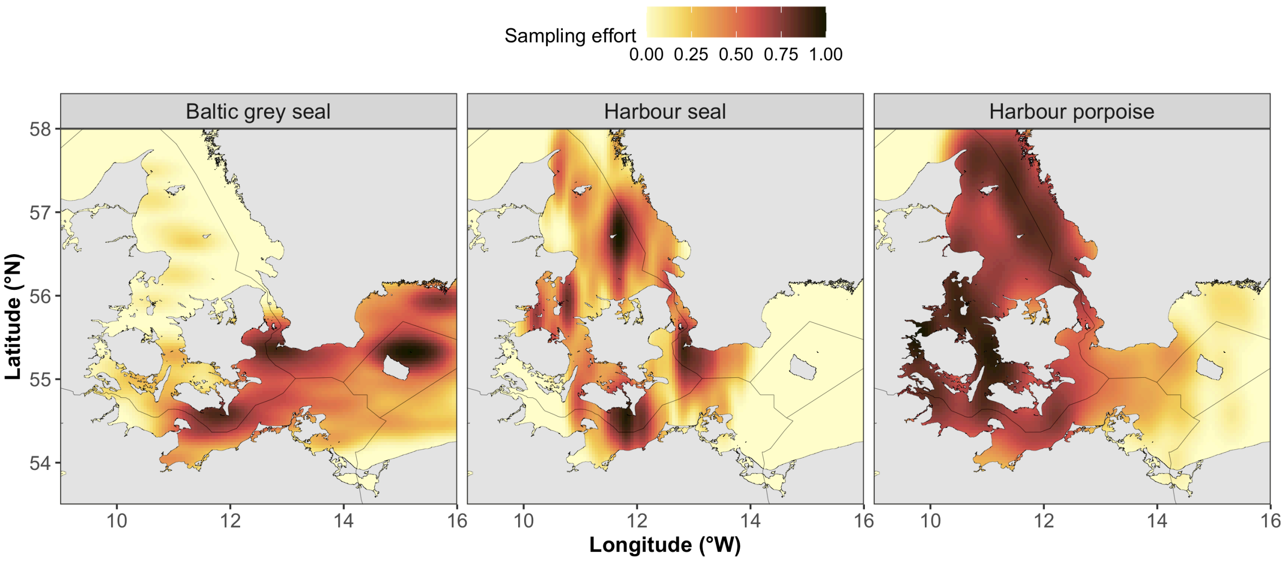

Figure S2.4: Species-specific Gaussian kernel density rasters of all sampling locations.

Table S2.1. Results of the species-specific pruning of MaxEnt models based on AUC and OR10 using location data collected in the southwestern Baltic Sea during 1997-2020. Through the R package ‘ENMeval’, different settings of regularization multipliers (RM) (0.5 – 5.0 at 0.5 intervals) and feature classes (‘linear’, ‘quadratic’, ‘linear & quadratic’) were tested. Presented are selected ‘optimal’ allowed feature classes and RM, as well as the mean and standard deviation (sd) of the area under the receiver operating characteristic curve (AUC), the mean and sd of ‘10% training omission rate’ (OR10) and the degrees of freedom (df) for each model.

	**Baltic grey seal**	**Harbour seal**	**Harbour porpoise**
**Feature class**	Q	LQ	LQ
**RM**	3.5	0.50	0.50
**AUC mean**	0.71	0.722	0.731
**AUC sd**	0.005	0.017	0.015
**OR10 mean**	0.099	0.098	0.099
**OR10 sd**	0.030	0.032	0.014
**df**	8	14	12

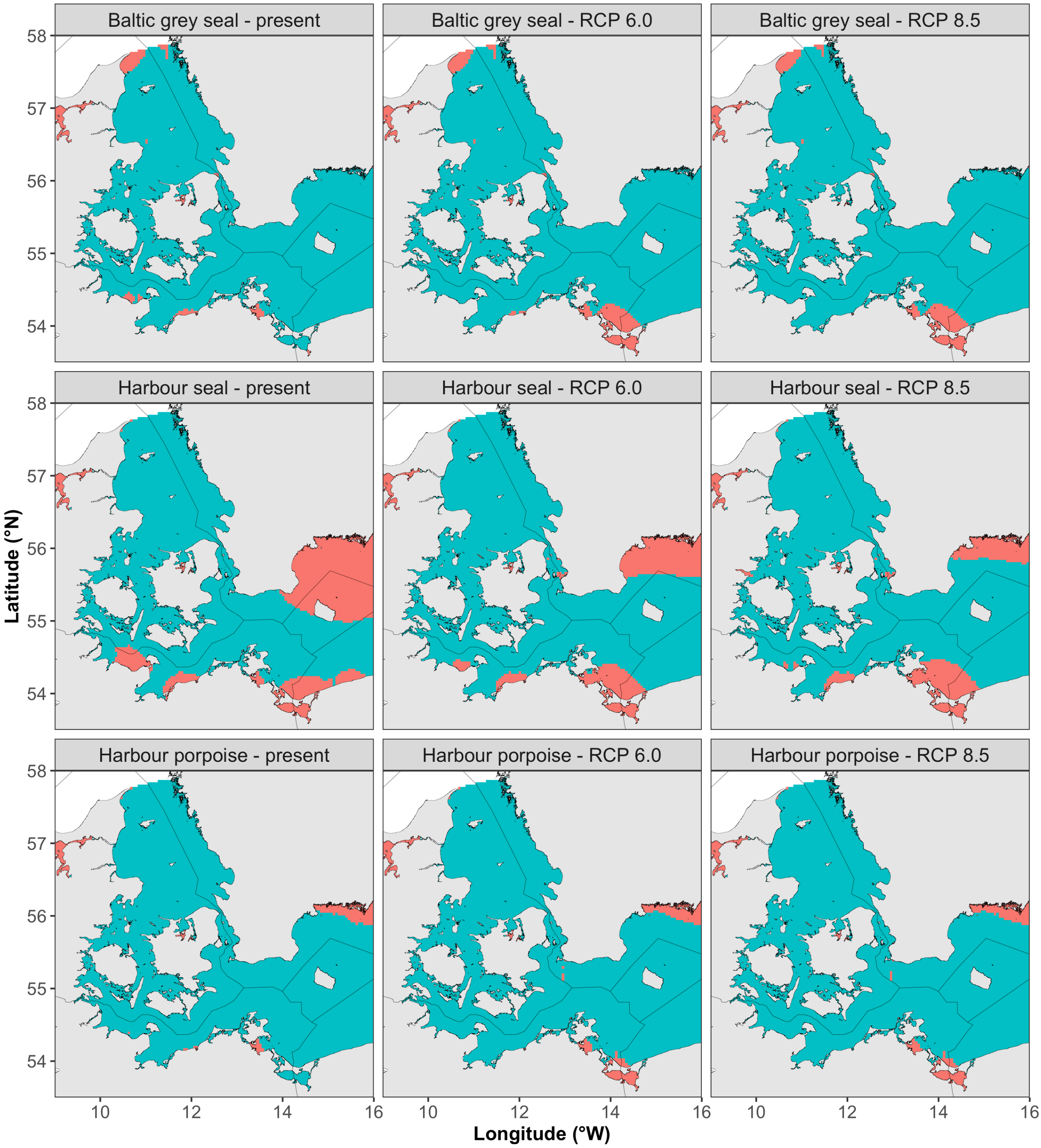

Figure S2.5: Results of the species-specific MESS analysis showing similarity between current (1997-2020) and future (2090-2100) conditions under the RCP 6.0 and 8.5 scenario for the study area. Predictions of habitat suitability as derived by MaxEnt were subsequently restricted (i.e. masked) to pixels with a similarity value of >0.

Table S2.2. Mean (SD) threshold values derived from the species-specific optimal MaxEnt models.

Species	Kappa	MSSS	P10
Baltic grey seal	0.779 (0.008)	0.598 (0.008)	0.079 (0.003)
Harbour seal	0.732 (0.024)	0.594 (0.005)	0.107 (0.009)
Harbour porpoise	0.705 (0.021)	0.542 (0.006)	0.168 (0.018)

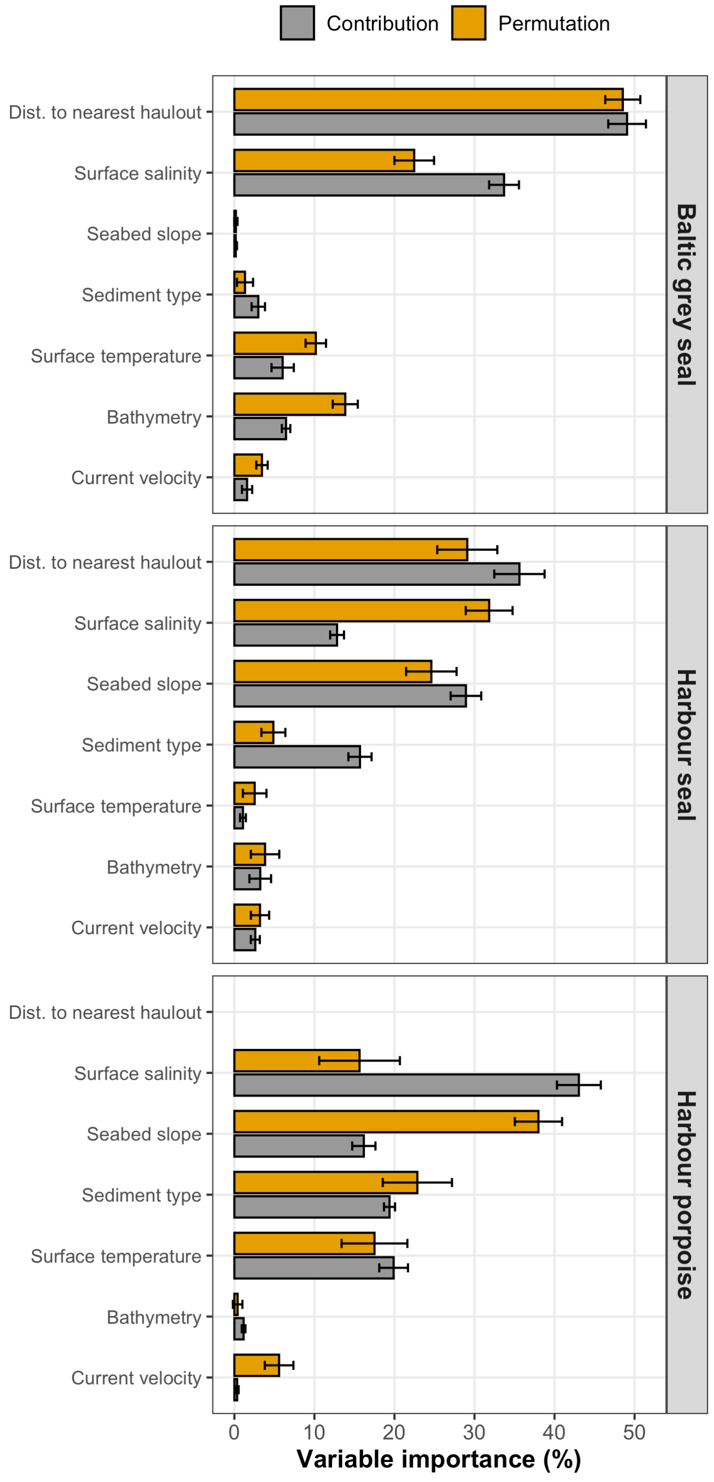

Figure S2.6: Results of the variable importance assessment showing the mean permutation importance (orange bars) and percent contribution (grey bars) of each predictor variable included in the species-specific MaxEnt models as identified by OR10 and AUC using location data collected in the southwestern Baltic Sea during 1997-2020. Ten replicates of each full model were run to generate the mean and 95% confidence intervals.

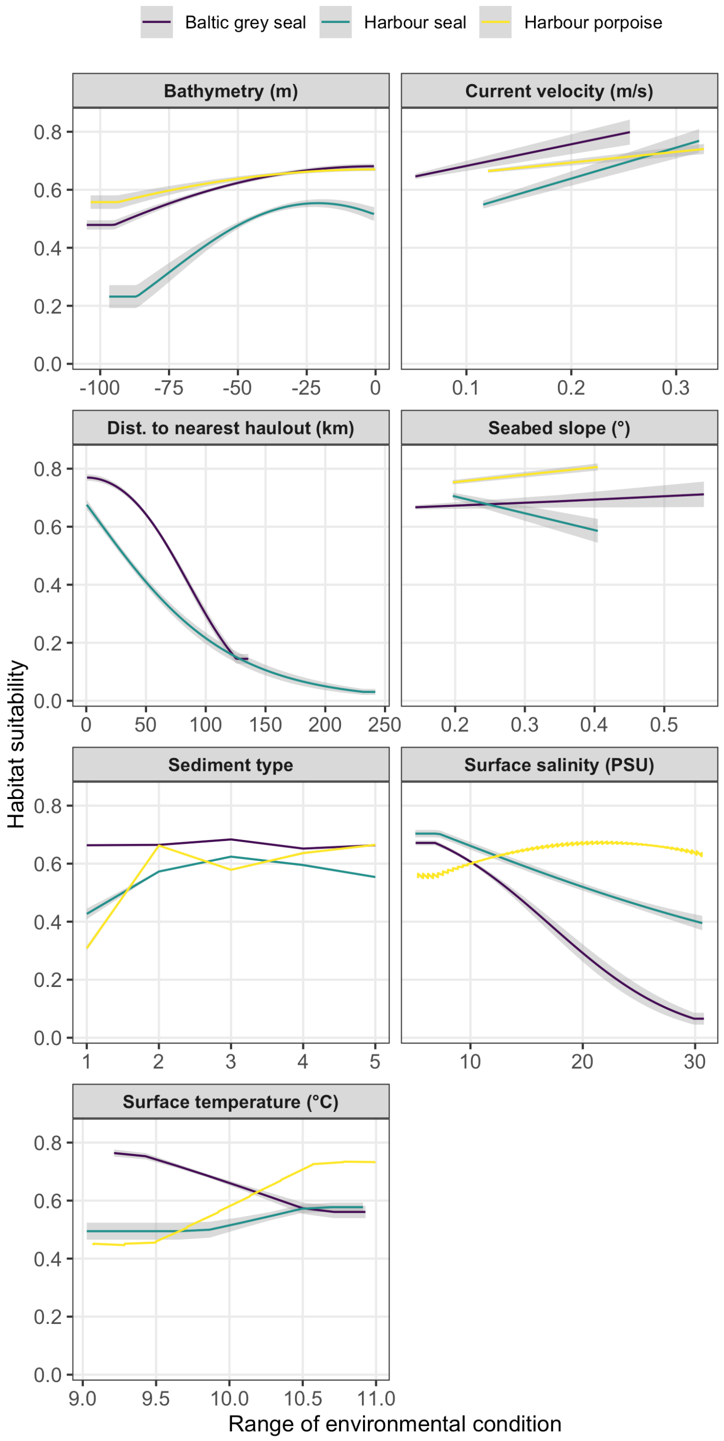

Figure S2.7: Overview of the response curves of the optimal species-specific MaxEnt models as evaluated by OR10 and AUC using location data collected in the southwestern Baltic Sea during 1997-2020. Each curve shows how habitat suitability changes across the environmental gradient while keeping the other predictor variables constant at their median value. Ten replicates of each model were run to generate the mean 95% confidence intervals.
